# Revealing the microbial assemblage structure in the human gut microbiome using latent Dirichlet allocation

**DOI:** 10.1186/s40168-020-00864-3

**Published:** 2020-06-23

**Authors:** Shion Hosoda, Suguru Nishijima, Tsukasa Fukunaga, Masahira Hattori, Michiaki Hamada

**Affiliations:** 1 Graduate School of Advanced Science and Engineering, Waseda University, 55N-06-10, 3-4-1, Okubo Shinjuku-ku, Tokyo, 169–8555 Japan; 2 Computational Bio Big-Data Open Innovation Laboratory (CBBD-OIL), National Institute of Advanced Industrial Science and Technology (AIST), Tokyo, Japan; 3 Computational Biology and Medical Sciences, Graduate School of Frontier Sciences, The University of Tokyo, Chiba, Japan; 4 Department of Computer Science, Graduate School of Information Science and Engineering, The University of Tokyo, Tokyo, Japan; 5 RIKEN Center for Integrative Medical Sciences, Kanagawa, Japan; 6 Graduate School of Medicine, Nippon Medical School, Tokyo, Japan; 7 Center for Data Science, Waseda University, Tokyo, Japan

**Keywords:** Metagenomics, Latent Dirichlet allocation, Human gut microbiome, Enterotype, Microbial assemblage, Bayesian model, Machine learning

## Abstract

**Background:**

The human gut microbiome has been suggested to affect human health and thus has received considerable attention. To clarify the structure of the human gut microbiome, clustering methods are frequently applied to human gut taxonomic profiles. Enterotypes, i.e., clusters of individuals with similar microbiome composition, are well-studied and characterized. However, only a few detailed studies on assemblages, i.e., clusters of co-occurring bacterial taxa, have been conducted. Particularly, the relationship between the enterotype and assemblage is not well-understood.

**Results:**

In this study, we detected gut microbiome assemblages using a latent Dirichlet allocation (LDA) method. We applied LDA to a large-scale human gut metagenome dataset and found that a 4-assemblage LDA model could represent relationships between enterotypes and assemblages with high interpretability. This model indicated that each individual tends to have several assemblages, three of which corresponded to the three classically recognized enterotypes. Conversely, the fourth assemblage corresponded to no enterotypes and emerged in all enterotypes. Interestingly, the dominant genera of this assemblage (*Clostridium*, *Eubacterium*, *Faecalibacterium*, *Roseburia*, *Coprococcus*, and *Butyrivibrio*) included butyrate-producing species such as *Faecalibacterium prausnitzii*. Indeed, the fourth assemblage significantly positively correlated with three butyrate-producing functions.

**Conclusions:**

We conducted an assemblage analysis on a large-scale human gut metagenome dataset using LDA. The present study revealed that there is an enterotype-independent assemblage.

Video Abstract

## Introduction

The human gut microbiome varies greatly from person to person depending on differences among human populations [1] and dietary habits [2]. The differences in gut microbial compositions affect host health and physiology [3], and in some cases, altered microbial compositions are associated with diseases, such as inflammatory bowel disease (IBD) [4], type 1 diabetes [5], colorectal cancer [6], and autism [7, 8]. Recent developments in metagenome sequencing technologies have enabled investigations of gut microbial compositions of individuals with ease and rapidity, and many large-scale research projects focused on the human gut microbiome have been conducted [1, 9–11]. At present, by applying various data mining methods to these massive metagenomic datasets, the structure of the human gut microbiome and the relationship between a hosts phenotype and its gut microbial profile can be revealed.

**Fig. 1:**
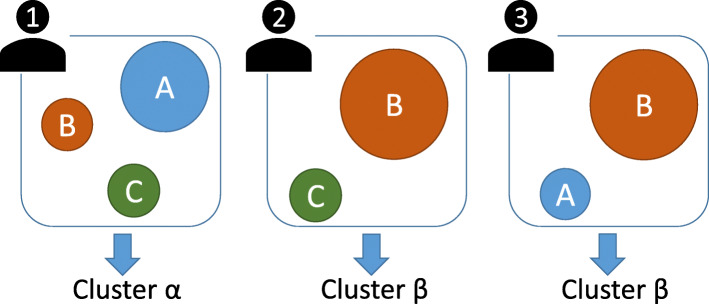
Schematic illustration of microbial assemblages and cluster analysis for the human gut microbiome, where A, B, and C indicate microbial assemblages, with circle size indicating abundance. The cluster of each individual is determined by the dominant assemblage. However, a cluster analysis cannot capture the non-dominant but shared microbes among samples, such as those comprising assemblage C

Cluster analysis of samples is one of the widely used data-mining methods in metagenomic research. With this approach, individuals are clustered into groups based on similarities in their microbial profiles, that is, each sample is assigned to one cluster by this method. For example, Arumugam et al. discovered that the gut microbial profiles of individuals could be classified into three types known as enterotypes using the partitioning around medoids (PAM) clustering method [12]. In another example, Ding and Schloss reported, employing the Dirichlet multinomial mixture (DMM) clustering method, that the human gut microbiome has considerable inter-individual variation and that the cluster type of an individual was almost unchanged during the sampling period [13, 14]. Although cluster analysis is a powerful approach for uncovering the overall structure of the human gut microbiome, this analysis is strongly affected by the dominant microbes in each individual. Therefore, cluster analyses of samples may ignore the existence of non-dominant but shared microbes among individuals (Fig. 1).

An alternative data-mining method is microbial assemblage analysis, which clusters microbes into certain assemblages, instead of clustering samples into groups. Here, following Shafiei et al. [15], we define microbial assemblages as groups of microbes that are expected to co-occur. The existence of microbial assemblages can be reasonably expected from the interactions between microbes [16]. Several microbial assemblages can exist in one individual, and microbial assemblage analysis can capture assemblages consisting of non-dominant microbes, unlike a cluster analysis of samples (Fig. 1). Shafiei et al. developed BioMiCo, which is a Bayesian probabilistic model for microbial assemblage analysis, and discovered host-specific assemblages in human gut metagenomic time-series data [15]. Cai et al. also explored microbial assemblages using non-negative matrix factorization methods and identified a shift of microbial assemblages through time in one individual [17]. Higashi et al. developed Latent Environment Allocation, a web application for visualization of metagenomic data based on a microbial assemblage analysis method, and found that microbial assemblages can represent continuous variations of the human gut microbiome [18]. Meanwhile, Yan et al. created MetaTopics, an R package for microbial assemblage analysis [19]. Further, Sankaran and Holmes conducted a simulation study to compare several methods of microbial assemblage analyses [20]. Microbial assemblage analysis of the human gut microbiome has also been applied to track sources of contamination in metagenomic research [21] and detect assemblage-level metabolic interactions [22]. Although many microbial assemblage analyses of the human gut microbiome have been performed, a comparison between classical enterotypes and the assemblages of co-occurring taxa has not yet been conducted. Consequently, the large-scale assemblage structure of the human gut microbiome and the relationship between microbial assemblages and enterotypes are still unclear.

In this study, we carried out a microbial assemblage analysis of a large-scale human gut metagenomic dataset to establish the relationship between microbial assemblages and enterotypes. To detect assemblages, we used the latent Dirichlet allocation (LDA) method, which is an unsupervised probabilistic model [23]; LDA was first proposed for the classification of documents in natural-language processing, and this method is now widely applied in bioinformatics fields, such as transcriptome analysis [24], pharmacology [25], gene function prediction [26], and metagenomic analyses [18–20, 27]. We first investigated the number of microbial assemblages based on the relationship between microbial assemblages and enterotypes. We found that a 4-assemblage model has high interpretability in the context of a large-scale human gut microbiome dataset and discovered that an individual might have not just one microbial assemblage but several assemblages in many cases. We investigated the relationships between enterotypes and microbial assemblages and revealed that three of the assemblages could be matched to the three enterotypes; however, the fourth assemblage could exist in all enterotypes. In addition, the dominant genera of this assemblage included butyrate-producing species, and this assemblage is significantly positively correlated with three butyrate-producing functions. We also estimated the functions of each assemblage by applying LDA to the functional profiles of the same samples and found that the fourth assemblage has some specific functional categories, such as the immune system and translation, with high abundance.

## Materials and methods

We used our own implementation of the LDA and PAM algorithms. The detailed information is described in “Availability of data and material.”

### Metagenomic dataset and preprocessing methods

We used the large-scale human gut metagenome dataset constructed by Nishijima et al. [28]. This dataset consisted of gut metagenomic data from 861 healthy adults from 12 countries. Each individual corresponds to one sample. The taxon of each sequencing read was assigned by mapping the read to a reference genome dataset consisting of 6149 microbial genomes. We used genus as the taxonomic rank for each sequencing read, as commonly performed in previous studies on enterotypes [12–14, 29].

The read count of each genus of each individual was divided by the total read count of each individual, then multiplied by 10,000 and rounded down to the nearest integer. This is because the estimation results of LDA are strongly affected by samples with high read counts when using read count data directly. Therefore, we normalized each sample to sum to the same constant (that is, 10,000) to remove the bias caused by the differences of read counts among samples. We confirmed that the estimated parameters do not depend on the constant (Additional File 1: Figure S1). After these preprocessing steps, the number of different genera included in the dataset became 252.

To calculate correlation coefficients between microbial assemblages and functions across individuals, we used the Kyoto Encyclopedia of Genes and Genomes (KEGG) [30] orthology-based annotated data as functional profiles. These functional profiles are of the same samples as the genus data.

### PAM clustering method

To assign enterotypes, we applied the PAM clustering method to the dataset according to the methods of Arumugam et al. [12]. This algorithm clusters samples by iteratively updating each cluster’s medoid, which is defined as the sample in a cluster for which the sum of a dissimilarity to the other samples in the same cluster is the smallest. The algorithm consists of the following three steps: (*i*) choose the initial value of the medoid randomly from the samples, (*ii*) assign each sample to the cluster with the smallest Jensen–Shannon divergence (JSD) to its medoid, and (*iii*) update the medoids using the JSD as the dissimilarity. Repeat steps (*ii*) and (*iii*) until the medoids no longer change. In the present study, we conducted 10 trials and used the result that had the highest silhouette coefficient [31].

### LDA for modeling the human gut microbiome

The probabilistic LDA model [23] can be utilized to estimate *K* microbial assemblages from a human gut metagenomic dataset, where *K* is a given parameter. Let the numbers of individuals (samples) and genera be denoted by *N* and *D*, respectively. In the LDA model, the *i*th metagenome sample (*i*∈{1,...,*N*}) has a categorical distribution with parameter $\theta _{i}=\{\theta _{i,k}\}_{k=1}^{K}$ over microbial assemblages where *θ*_*i*,*k*_ is the occurrence probability of the *k*th assemblage in the *i*th sample. The *k*th microbial assemblage has a categorical distribution with parameter $\phi _{k}=\{\phi _{k,j}\}_{j=1}^{D}$ over genera, where *ϕ*_*k*,*j*_ is the occurrence probability of the *j*th genus in the *k*th assemblage.

A microbial assemblage with high probability in an individual implies that the individual tends to have that particular microbial assemblage in the gut microbiome, and a genus with high probability in a microbial assemblage indicates that the microbial assemblage tends to have that particular genus. In addition, the LDA model has prior distributions on *θ*_*i*_ and *ϕ*_*k*_ provided by the Dirichlet distribution with hyperparameters *α* and *β*, respectively. In this study, we used 0.1 and 0.05 as initial values for all the elements in **α** and **β**, respectively.

The LDA parameters (*θ* and *ϕ*) can be learned from the dataset in an unsupervised manner. Various parameter inference methods for the LDA model have been proposed, and we used the variational Bayes (VB) method [23]. The VB method maximizes an approximation of the marginal likelihood, called the variational lower bound (VLB) score, by updating the parameters iteratively from random initial values. We finished the iteration of the parameter update when the change in the VLB score between the previous and the current step was less than 10^−6^. Finally, we estimated each *θ*_*i*_ and *ϕ*_*k*_ as the expectation values of the posterior distribution estimated by the VB method. This parameter estimation method has been previously described by Asuncion et al. [32]. In addition, we updated the hyperparameters **α** and **β** from the initial values using a fixed-point iteration method in the parameter learning step [33]. Based on previous research on LDA hyperparameter settings [34], we estimated the parameters such that each element of **α** differed from the others but all elements of **β** had the same value. We conducted 10 trials for each *K*=2, 3, 4, and 5 and adopted the estimated set of parameters with the highest VLB score among all trials for each *K*.

### Functional assemblage analysis

We estimated the functions of each assemblage by applying LDA to the functional profiles. We refer to the resulting assemblages as *functional assemblages*. Details surrounding the method are described in Section S1 (Additional File 1).

### Entropy scores of genera and individuals

To quantify whether the estimated distributions are skewed toward some assemblages, we calculated the entropy scores of each sample and each genus over assemblages. In a categorical distribution, a high entropy score means that the distribution is similar to the uniform distribution, and a low score means that the distribution tends to take a specific value. The entropy score *H*(*i*) of the *i*th sample over assemblages was calculated as follows:

1 $$ H(i) = -\sum_{k=1}^{K} P(k|i) \log P(k|i).   $$

As *P*(*k*|*i*) is equal to *θ*_*i*,*k*_, we can directly calculate this score using the estimated LDA parameters. The entropy score, *H*(*j*), of the *j*th genus over assemblages was calculated as follows:

2 $$ H(j) = -\sum_{k=1}^{K} P(k|j) \log P(k|j),   $$

3 $$ P(k|j) = \frac{P(j|k) P(k)}{\sum_{k} P(j|k) P(k)}   $$

where *P*(*j*|*k*) is equal to *ϕ*_*k*,*j*_ and *P*(*k*) was computed as the average of all *θ*_*i*,*k*_ across samples.

## Results

### Cluster analysis of the human gut microbiome enterotypes

To investigate the relationship between enterotypes and assemblages, we classified individual samples into three clusters using the PAM clustering method. We observed that the dominant genera in each identified cluster were *Bacteroides*, *Prevotella*, and *Blautia* and that these genera were specific to each cluster (Additional File 1: Figure S2). These results were consistent with those of previous enterotype studies, in which the following three enterotypes were identified in the human gut microbiome: *Bacteroides* dominant type, *Prevotella* dominant type, and *Ruminococcus* and *Blautia* dominant type [12]. Hence, we referred to these clusters as B-type, P-type, and R-type. These results were consistent across trials (Additional File 1: Figure S3)

### Analysis of the human gut microbial assemblage profiles estimated by LDA

**Fig. 2:**
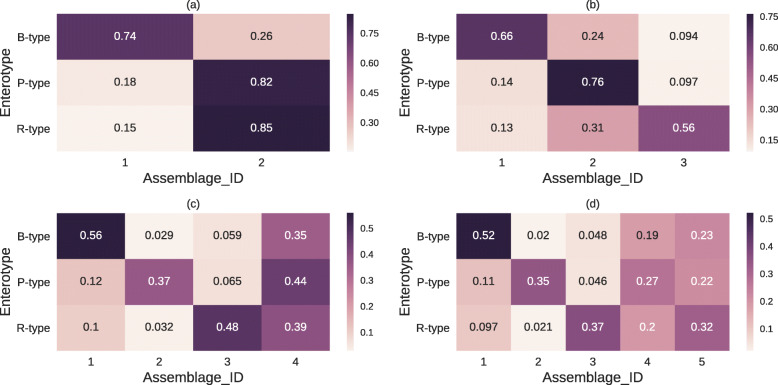
Assemblage distributions for each enterotype. Each row shows a distribution obtained by averaging the estimated assemblage distributions across individuals of each enterotype. The *x*- and *y*-axes represent the microbial assemblages and enterotypes, respectively. Darker colors indicate higher probabilities, and each number inside the partition indicates a different probability, where the sum of the values over each row is 1. **a**, **b**, **c**, and **d** indicate *K*-assemblage LDA models with *K*=2,3,4, and 5, respectively

We estimated the *K*-assemblage LDA model parameters for *K*=2,3,4, and 5 to identify the model with the highest interpretability of relationships between enterotypes and assemblages. Figure 2 shows the assemblage distributions for each enterotype obtained by each model for each assemblage (the standard deviations of the distribution across individuals are shown in Additional File 1: Figure S4). The 2-assemblage model identified a B-type specific assemblage and a P- and R-type specific assemblage (IDs 1 and 2 in Fig. 2a). The 3-assemblage model estimated assemblages corresponding to each enterotype (Fig. 2b). In addition to these enterotype-specific assemblages, the 4- and 5-assemblage models inferred general assemblages that appear in *all* the enterotypes (Fig. 2cd). The strength of LDA is that it is possible to obtain such an assemblage. Adding a fifth assemblage is not informative because it yields two general assemblages (IDs 4 and 5 in Fig. 2d) that have the same abundance pattern for enterotypes. Therefore, we used the 4-assemblage model in this study. We emphasize that the existence of a general assemblage is not trivial in models with four or more assemblages because there are not always genera that appear in all enterotypes. These results are consistent across trials (Additional File 1: Figure S5)

**Fig. 3:**
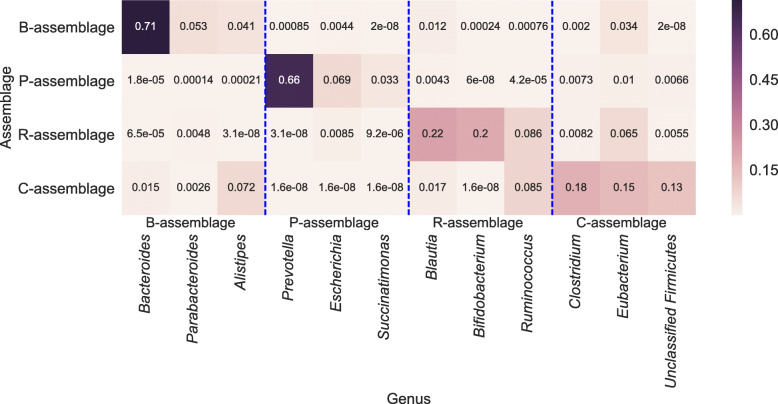
Estimated genus distribution of each microbial assemblage. The *x*- and *y*-axes represent genera and assemblages, respectively. We displayed only the three genera with the highest probability in each assemblage

In the following analysis, we call the assemblages with IDs 1, 2, and 3 the “B-assemblage,” “P-assemblage,” and “R-assemblage,” respectively, because these assemblages appeared specifically in the B-, P-, and R-type individuals, respectively (Fig. 2c). In addition, we refer to the assemblage with ID 4 as the “C-assemblage” owing to the high proportion of *Clostridium*. Next, we investigated the taxonomic composition of each microbial assemblage. Figure 3 depicts the genus distribution of each microbial assemblage estimated by LDA (i.e., $\phi _{k}=\{\phi _{k,j}\}_{j=1}^{D}$ in the previous section). B- and P-assemblages mainly consisted of one dominant genus, *Bacteroides* and *Prevotella*, with relative frequencies of 71% and 66%, respectively. Conversely, R- and C-assemblages consisted of genera with moderate abundance. The genera that constituted the R-assemblage were *Blautia* (22%), *Bifidobacterium* (20%), and *Ruminococcus* (8.6%). The C-assemblage consisted of *Clostridium* (18%), *Eubacterium* (15%), and unclassified *Firmicutes* (13%).

**Fig. 4:**
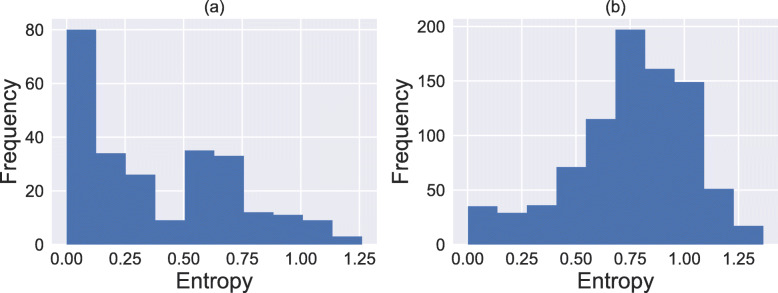
Histograms of the entropy scores over assemblages **a** for all genera (Eq. 2) and **b** for all individuals (Eq. 1). The *x*- and *y*-axes represent entropy and the number of samples, respectively

[In the LDA model, a genus can appear in several microbial assemblages. We investigated whether genera occurred in just one specific assemblage or not using the entropy scores of genera over assemblages (Eq. 2). Figure 4a shows a histogram of the entropy scores for all genera, and two peaks at 0.00–0.125 and 0.50–0.75 can be observed within the distribution. The former peak represents assemblage-specific genera, and *Bacteroides* and *Prevotella* belonged to this group (Additional File 1: Table S1). The latter peak represents a genus appearing in several, but not all, assemblages, and *Ruminococcus* and *Blautia* belonged to this group (Additional File 1: Table S1). Several genera had high entropy scores, thereby indicating that they are universal genera among assemblages (Additional File 1: Figure S6).

Next, we calculated the entropy score of individuals over assemblages (Eq. 1). The distribution of the entropy scores was unimodal (Fig. 4b), and the median was 0.7805. These results suggest that most individuals have multiple, but not all, microbial assemblages. In addition, we examined the distribution of assemblages within individuals (Additional File 1: Figure S7) and found that the co-abundance tendencies between microbial assemblages were not uniform. Individuals with B-assemblage, P-assemblage, R-assemblage, and C-assemblage dominance tend to have neither the P- nor R-assemblage, not the R-assemblage, not the P-assemblage, and possess any other assemblages, respectively. All the individuals tend to have the C-assemblage. These co-abundance tendencies can occur in the case that there are actually four enterotypes and one corresponding assemblage for each. To investigate such a possibility, we performed 4-type PAM clustering, but no such one-to-one relationship was observed (Additional File 1: Figure S8). Therefore, the C-assemblage can be regarded as an assemblage that appears in all three enterotypes.

### Relationships between microbial assemblages and countries

**Fig. 5:**
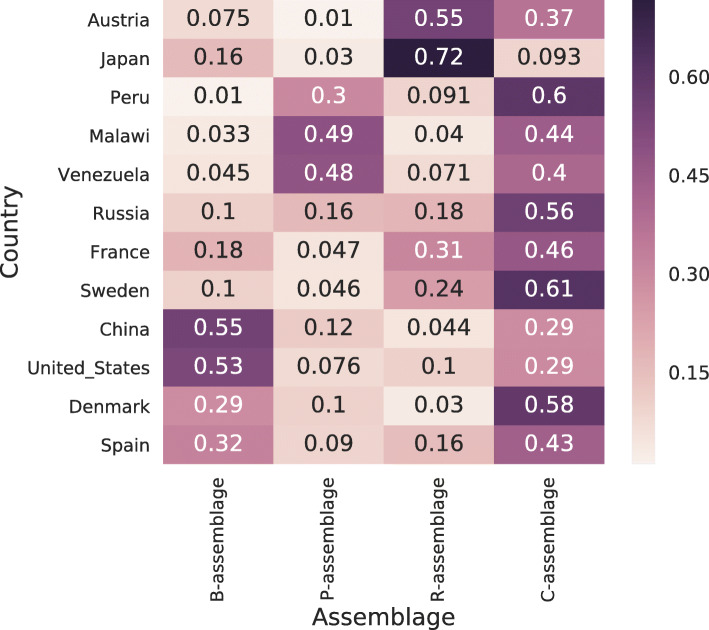
Average assemblage distributions for each country. Each row shows a distribution obtained by averaging the estimated assemblage distributions across individuals of each country. The *x*- and *y*-axes represent the microbial assemblage and country of the individual, respectively

We investigated the relationship between microbial assemblages and host countries. Figure 5 shows the average assemblage distributions of individuals for each country (the standard deviations of the distribution across individuals are shown in Additional File 1: Figure S9). We discovered that the occurrence distributions of microbial assemblages vary from country to county; for example, Japan and Austria tend to have R-assemblages while Peru, Malawi, and Venezuela tend to have P-assemblages. Conversely, the C-assemblage was frequently found in all countries except Japan.

### Correlations between microbial assemblages and butyrate-producing functions

**Fig. 6:**
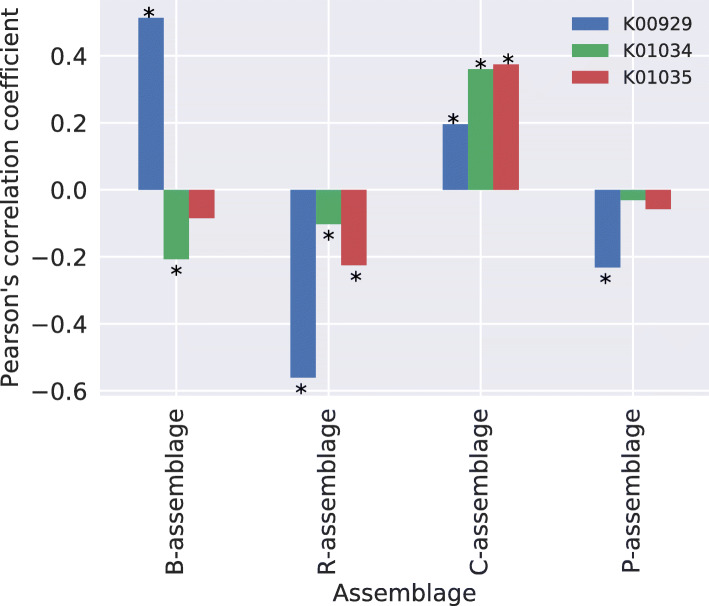
The Pearson’s correlation coefficients among the four assemblages and three butyrate-producing functions. The *x*- and *y*-axes represent the assemblages and Pearson’s correlation coefficients, respectively. Each bar of each assemblage indicates, from left to right, K01034, K00929, and K01035, respectively. Asterisks indicate significant differences. Significance was determined at *p*<0.01 (two-sided test, after Benjamini–Hochberg correction)

Dominant genera in the C-assemblage included butyrate-producing bacteria (Table 1). Thus, we examined correlations between microbial assemblages and butyrate-producing functions (K00929: butyrate kinase, K01034: acetate CoA/acetoacetate CoA-transferase alpha subunit, and K01035: acetate CoA/acetoacetate CoA-transferase beta subunit). Figure 6 indicates the Pearson’s correlation coefficients between microbial assemblages and butyrate-producing functions across individuals, showing that the C-assemblage is significantly positively correlated with all three functions (*p*<0.01, two-sided test, after Benjamini–Hochberg correction). The P- and R-assemblages were negatively correlated with some functions, and the B-assemblage was significantly positively correlated with only K00929, concurrent with the finding that *Bacteroides fragilis* has only K00929 among these three functions [35].

**Table 1:** Dominant genera of the C-assemblage an their probabilities as estimated by LDA

**Genus**	**Probability in C-assemblage**
*Clostridium*	0.179865
*Eubacterium*	0.150802
*Unclassified Firmicutes*	0.129783
*Faecalibacterium*	0.093720
*Ruminococcus*	0.085272
*Roseburia*	0.074214
*Alistipes*	0.072359
*Coprococcus*	0.029497
*Butyrivibrio*	0.021738

### Functional profiles of each microbial assemblage

To discuss the functional profiles of the microbial assemblages, we applied LDA to the functional profiles of individuals using the same *K* number as for the taxonomic profiles. We regarded functional assemblages as functional profiles of the microbial assemblages. More information on this experiment is described in the supplementary section (Additional File 1: Section S1). We obtained functional assemblages with a one-to-one correspondence with the estimated microbial assemblages (Additional File 1: Figure S10). These results justify regarding functional assemblages as functional profiles of the microbial assemblages. Next, we determined the abundances of functional categories for each assemblage (Additional File 1: Figure S11) and assessed the assemblages with the largest relative abundance for each functional category (Table 2). This table shows that metabolic functions of glycan/lipid are abundant in the B-assemblage and that some specific functional categories, such as the immune system and translation, are abundant in the C-assemblage. However, Supplementary Figure S11 demonstrates that differences between assemblages are rather small.

**Table 2:** Functional assemblage having the largest relative abundance for each functional category

**Functional category**	**Functional assemblage**
Biosynthesis of other secondary metabolites	B-assemblage(ko)
Carbohydrate metabolism	B-assemblage(ko)
Lipid metabolism	B-assemblage(ko)
Transport and catabolism	B-assemblage(ko)
Digestive system	B-assemblage(ko)
Endocrine system	B-assemblage(ko)
Glycan biosynthesis and metabolism	B-assemblage(ko)
Environmental adaptation	B-assemblage(ko)
Energy metabolism	P-assemblage(ko)
Endocrine and metabolic diseases	P-assemblage(ko)
Immune diseases	P-assemblage(ko)
Infectious diseases	P-assemblage(ko)
Metabolism of other amino acids	P-assemblage(ko)
Metabolism of terpenoids and polyketides	P-assemblage(ko)
Nervous system	P-assemblage(ko)
Excretory system	P-assemblage(ko)
Folding, sorting, and degradation	P-assemblage(ko)
Transcription	R-assemblage(ko)
Amino acid metabolism	R-assemblage(ko)
Metabolism of cofactors and vitamins	R-assemblage(ko)
Membrane transport	R-assemblage(ko)
Cell communication	R-assemblage(ko)
Signaling molecules and interaction	R-assemblage(ko)
Xenobiotics biodegradation and metabolism	R-assemblage(ko)
Immune system	C-assemblage(ko)
Nucleotide metabolism	C-assemblage(ko)
Neurodegenerative diseases	C-assemblage(ko)
Substance dependence	C-assemblage(ko)
Replication and repair	C-assemblage(ko)
Signal transduction	C-assemblage(ko)
Cell motility	C-assemblage(ko)
Cell growth and death	C-assemblage(ko)
Translation	C-assemblage(ko)
Cardiovascular diseases	C-assemblage(ko)
Cancers	C-assemblage(ko)

Each functional assemblage is indicated by the name of the corresponding microbial assemblage with (ko) appended

## Discussion

In this study, we used LDA for the detection of microbial assemblages in population-scale human gut microbiome data and discovered four microbial assemblages. While three assemblages (B-, P-, and R-assemblages) specifically emerged in the corresponding enterotypes (B-, P-, and R-types), the C-assemblage was frequently observed in every enterotype. As conventional cluster analysis of the samples focuses on the dominant genus of a cluster and the differences among clusters, the existence of non-dominant but shared microbial assemblages among individuals may have been overlooked. The detection of the C-assemblage suggested that LDA is a powerful approach for revealing the assemblage structure in large metagenomic datasets.

We chose *K*, i.e., the number of assemblages, based on assemblage interpretability after comparing models with different numbers of assemblages. This task, called "model selection," is typically difficult for mixture models. Some methods for this task have been previously suggested [36, 37]. Yan et al. used cross-validation, which is a method that selects the model with the highest likelihood against the test data [19]. However, these methods tend to overestimate *K*, leading to difficulties in clarifying the association between enterotypes and assemblages. Indeed, Yan et al. estimated *K*=60, although the number of samples was less than in this study.

As the model we used is rather simple (that is, *K* is small), it might fail to adequately capture the structure of the data. Hence, we confirmed whether our results were consistent with the data in two ways. First, we verified that the relative abundance (Additional File 1: Figure S12) was consistent with the estimated assemblage distribution (Fig. 2c). Each genus was regarded as mainly appearing in the assemblage with the highest *P*(*k*|*j*), as defined by Eq. 3. These results were consistent with the estimated parameters shown in Fig. 2c. Second, we verified that most genera within the same assemblage were significantly positively correlated across samples based on the Spearman’s correlation coefficients across samples between the major genera of the B-, P-, R-, and C-assemblages (*p*<0.01, two-sided test, after Benjamini–Hochberg correction [38], Additional File 1: Figure S13). Some genera (i.e., *Eubacterium*, *Faecalibacterium*, *Dorea*, *Ruminococcus*, *Streptococcus*, and *Catenibacterium*) were significantly positively correlated with many genera in other assemblages. These results are in agreement with the fact that their *P*(*k*|*j*) is high for multiple assemblages (Additional File 1: Figure S14). For example, *Ruminococcus* has a positive correlation with the genera mainly appearing in the R-assemblage. Indeed, *Ruminococcus* has a high association with the R-assemblage even though its main assemblage is the C-assemblage.

As mentioned earlier, the genera mainly appearing in the B- and P-assemblages tend to occur in the B- and P-types, respectively. The genera specifically appearing in the B- and P-types were reported to have functions for metabolizing protein/animal fat and carbohydrates, respectively [29], and the genera mainly appearing in the B- and P-assemblages may consequently have the same functions. We could confirm that lipid metabolism functions were abundant in the B-assemblage through functional assemblage analysis. This result suggests that the B-assemblage in the human gut becomes dominant through a fat-rich diet. Similarly, the genera mainly appearing in the C-assemblage may have functions that do not correspond with dietary habits because they appeared in all enterotypes. This suggestion is concurrent with the finding that functions related to immune cells and translation are abundant in the C-assemblage. The assemblage distributions for each country also suggests a relationship between dietary habits and assemblage. Peru, Malawi, and Venezuela, where staple foods include corn, have high P-assemblage abundance. We could not establish a similarity in dietary habits between Japan and Austria though their distributions are similar.

The noticeable characteristic of Japan, i.e., low C-assemblage abundance, was observed. Nishijima et al. reported that the Japanese gut microbiome is characterized by the low abundance of *Clostridium* and unclassified *Firmicutes*, which are the main components of the C-assemblage (Table 1) based on the same dataset [28]. Japan has the highest abundance of the R-assemblage, which shares *Ruminococcus* and *Eubacterium* with the C-assemblage. Hence, the two assemblages may have similar metabolic functions. Incidentally, *Eubacterium* and *Faecalibacterium*, which are the abundant genera in the C-assemblage, were not less abundant in the Japanese population compared with that of other countries (Additional File 1: Figure S15).

There are two interesting points regarding the C-assemblage. First, it can coexist with all of the other three assemblages, which were found in almost all countries. Therefore, the genera mainly appearing in the C-assemblage are generalists in the human gut environment [39, 40]. While generalists can adapt to diverse environments, they are not specialized to particular environments unlike specialists. This difference in survival strategy may be the reason why the genera mainly appearing in the C-assemblage were not dominant in the human gut microbiome. It is therefore possible that the C-assemblage is the core gut microbiome [9, 41]. However, C-assemblage abundance is not consistent from person to person; as such, what determines the existence of C-assemblages in the gut microbiome is unclear. Second, the dominant genera of the C-assemblage (such as *Clostridium*, *Eubacterium*, *Faecalibacterium*, *Roseburia*, *Coprococcus*, and *Butyrivibrio*) include representative butyrate-producing species (Table 1) [42, 43]. In addition, we found that the C-assemblage correlates with the three butyrate-producing functions. Butyrate is known to have anti-inflammatory effects [44] and is associated with IBD, type-2 diabetes, and colorectal cancer [45–47]. Therefore, C-assemblage abundance may indicate the health of its hosts, although the dataset used in this study contained only healthy individuals. In addition, we found that the ages and body mass indices (BMIs) of hosts did not relate to the presence of the C-assemblage (Additional File 1: Figure S16). Further research is accordingly required, such as via comparisons of C-assemblage abundance between individuals with and without a disease.

We envision two future directions for applications of LDA to metagenomic data. The first is its application to more diverse datasets. Metagenomic data have been sampled from not only human guts but also various other environments, such as the atmosphere [48], ocean [49, 50], and soil [51]. Application of LDA to these data should help reveal the structure of microbial assemblages on a global scale [52]. For example, Sommeria-Klein et al. recently applied LDA to taxonomic profiles of a tropical forest soil DNA dataset to reveal spatial structures [53]. The second direction is the extension of the LDA model—LDA has high model extensibility. Indeed, many extended LDA models have been proposed for natural-language processing [54–57]. The application of these extended LDA models to metagenomic analysis is a fascinating research focus for further elucidation of microbial assemblage structure. For example, applying supervised topic models [58], which utilize label information to estimate assemblage structures, to patient metagenomic data could detect microbial assemblages related to disease. The pachinko allocation model [54], which models hierarchical assemblage structures, may be useful for revealing sub-assemblages within an assemblage. A transition in assemblage composition can be estimated from time-series data from the human gut microbiome [59] using the topic tracking model [57].

## Conclusions

In this study, we conducted a microbial assemblage analysis on a large-scale human gut metagenome dataset using LDA. We discovered that three assemblages specifically emerged in the corresponding enterotypes, but the C-assemblage was frequently observed in all three enterotypes. In addition, we revealed that the dominant genera of the C-assemblage include representative butyrate-producing species. Further elucidation of the function of the C-assemblage or investigation of the relationship between disease and the C-assemblage is an important research direction.

## Supplementary information

**Additional file 1** This file includes Section S1, Figures S1, S2, S3, S4, S5, S6, S7, S8, S9, S10, S11, S12, S13, S14, S15, S16, and Table S1.

## Abbreviations

LDA: Latent Dirichlet allocation
IBD: Inflammatory bowel disease
PAM: Partitioning around medoids
DMM: Dirichlet multinomial mixture
BMI: Body mass index
VB: Variational Bayes
VLB: Variational lower-bound
