# Supplementary material for: Revealing the microbial assemblage structure in the human gut microbiome using latent Dirichlet allocation
Source: Microbiome. 2020 Jun 23;8:95. doi: 10.1186/s40168-020-00864-3 (PMC7313204; doi:10.1186/s40168-020-00864-3)
Supplement: Supplementary file 2 — Additional file 1 This file includes Section S1, Figures S1, S2, S3, S4, S5, S6, S7, S8, S9, S10, S11, S12, S13, S14, S15, S16, and Table S1. [file 40168_2020_864_MOESM1_ESM.pdf]

# Supplementary Materials for "Revealing the microbial assemblage structure in the human gut microbiome using latent Dirichlet allocation"

Shion Hosoda, Suguru Nishijima, Tsukasa Fukunaga, Masahira Hattori and Michiaki Hamada *

## S1 Functional assemblage analysis

We investigated the human population-level differences in microbial assemblages and their compositions. We also estimated the functions of each assemblage by applying LDA to the functional profiles of identical samples as the genus data. These assemblages are referred to as "functional assemblages" in later analyses.

### S1.1 Functional profile dataset and preprocessing methods

We normalized the functional profiles in the same manner as for the taxonomic profiles in the main text. We confirmed that the estimated parameters do not depend on the constants (Figure A1). After these preprocessing steps, the number of KEGG orthology types included in the dataset was 4,840.

### S1.2 LDA for modeling the human gut functional profiles

The inference method for functional assemblages is almost the same as that for microbial assemblages (the details are described in the "Materials and methods" section in the main text), with differences in the following two points. First, we used the same number of functional assemblages with the microbial assemblages, *i.e.* $K = 4$. Second, we calculated the correlation score (the details are described in the next paragraph) for each trial and adopted the trial with the highest correlation score (see the next paragraph) among all 10 trials.

We defined the correlation score to quantify appropriateness of treating estimated functional assemblages as the relative abundance of functions of microbial assemblages. The correlation score is defined by

$$\max_{p} \sum_{k=1}^{4} C_{k,p_k}, \tag{1}$$

where $C_{k,k'}$ is a Pearson's correlation coefficient between the $k$-th microbial assemblage and $k'$-th functional assemblage with respect to samples, $p$ is a permutation of functional assemblage indices $\{1, 2, 3, 4\}$, and $p_i$ is the $i$-th element of the permutation, $p$ (that is, $1, 2, 3$, or $4$).

As a result of 10 trials, the third trial, which had the highest correlation score, was adopted (Figure A2). We named the functional assemblages based on the corresponding microbial assemblage with (ko) appended (that is, "B-assemblage(ko)," "P-assemblage(ko)," "R-assemblage(ko)," and "C-assemblage(ko)").

*To whom correspondence should be addressed. Department of Electrical Engineering and Bioscience Faculty of Science and Engineering, Waseda University 55N-06-10, 3-4-1, Okubo Shinjuku-ku, Tokyo 169-8555, Japan. Tel: +81 3 5286 3130; Fax: +81 3 5286 3130; Email: mhamada@waseda.jp

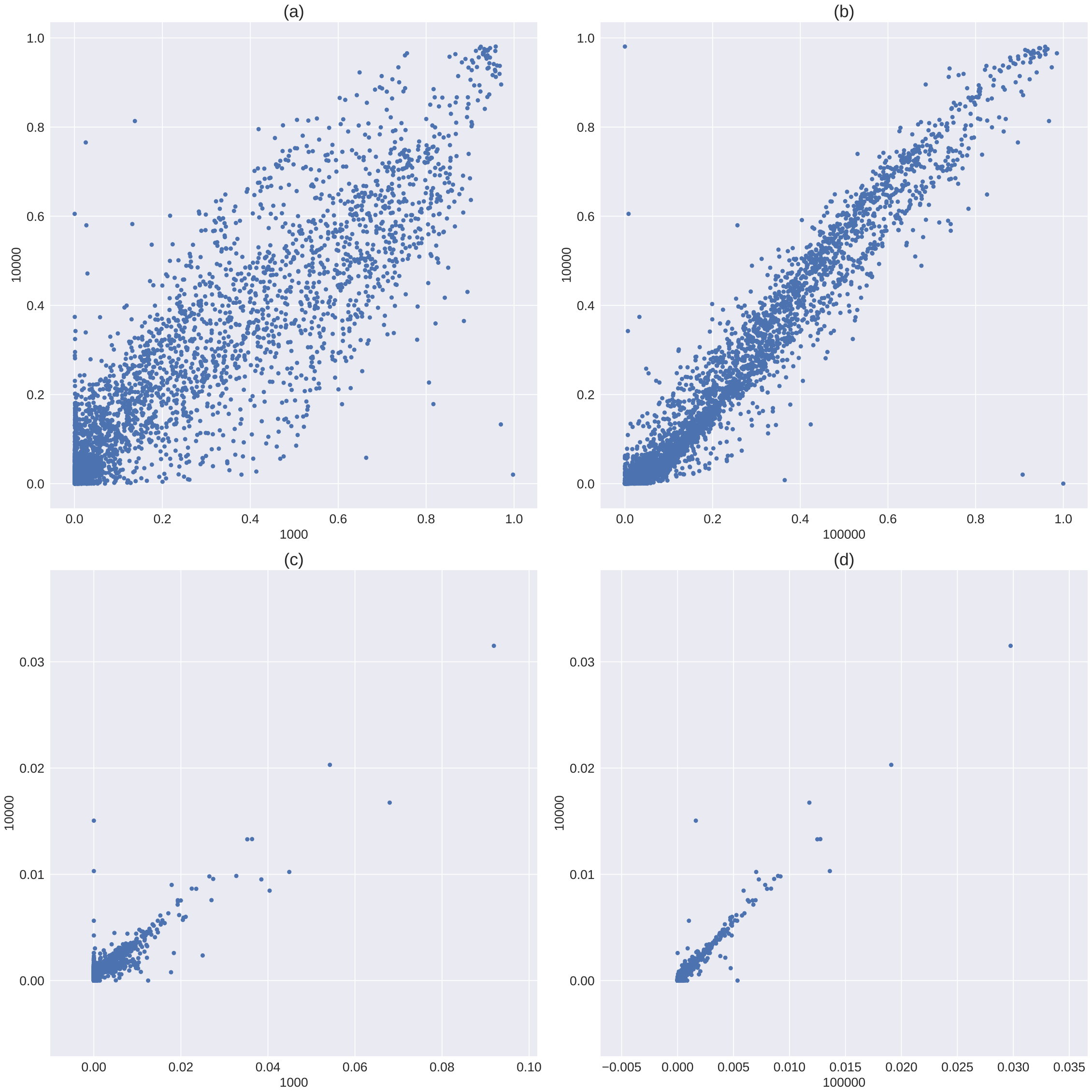


Figure A1: Scatter charts between estimated parameters of the constants (described in the main text) of 10,000 and 1,000/100,000. (a)(b) Parameters of the distributions of samples over functional assemblages. (a) 10,000 versus 1,000. (b) 10,000 versus 100,000. (c)(d) Parameters of the distributions of functional assemblage over functions. (c) 10,000 versus 1,000. (d) 10,000 versus 100,000. The $x$- and $y$-axes represent the estimated values of the parameters in the case of 1,000/100,000 and 10,000, respectively.

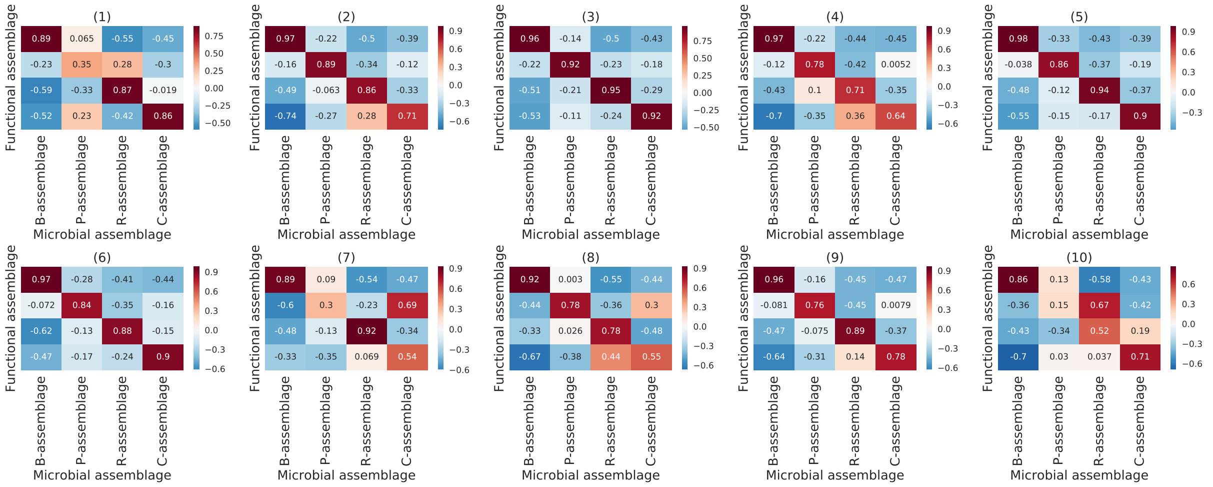

Figure A2: Pearson's correlation coefficients among microbial assemblages and functional assemblages for each trial ($C$ in section S1). Both the $x$- and $y$-axes represent assemblages.

## S2 Supplementary Figures and Table

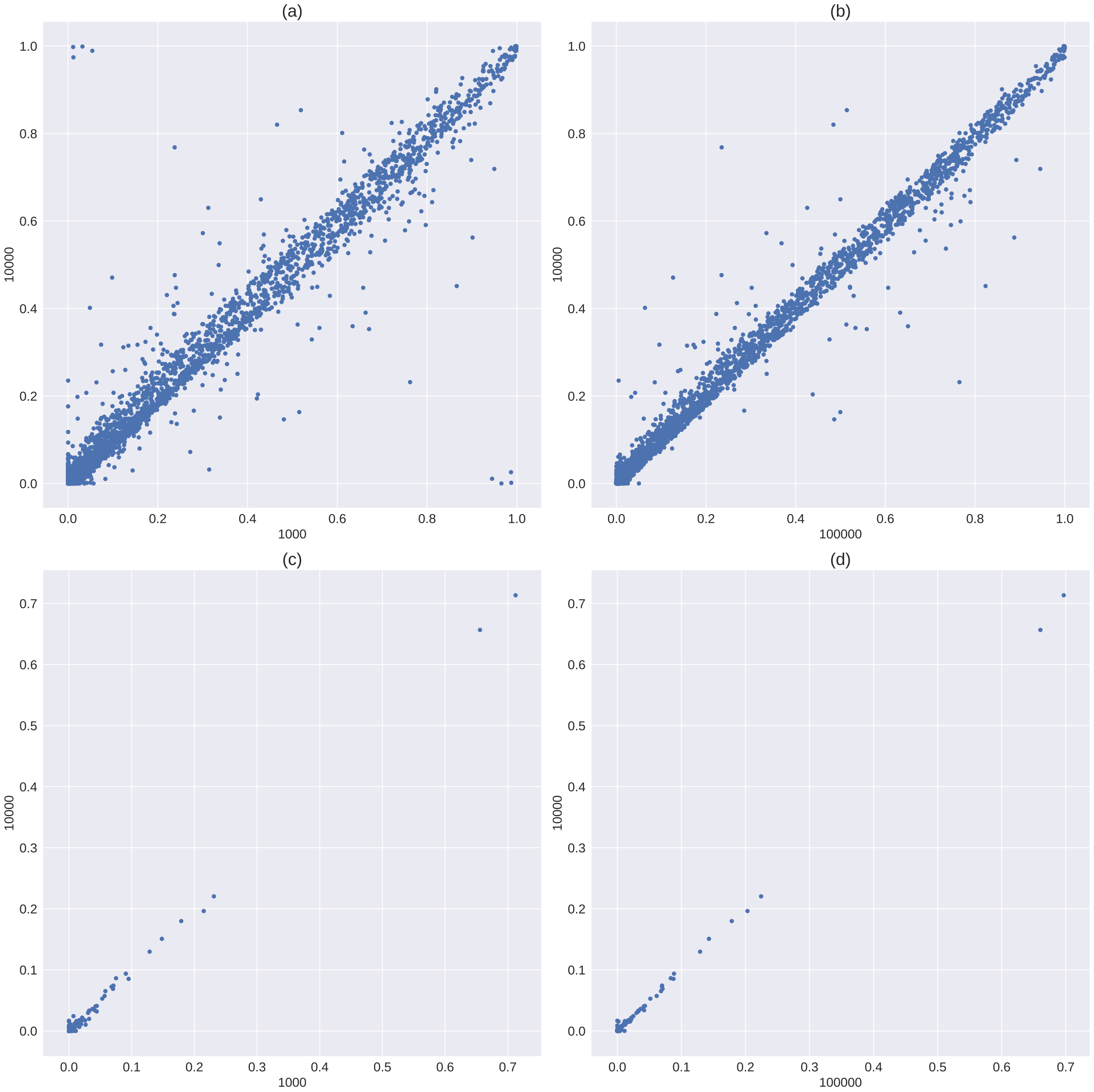


Figure S1: Scatter charts for estimated parameters with respect to different constants. (a,b) Comparison of estimated parameters of distributions of samples over microbial assemblages (indicated as $\theta_{i,k}$ in the main text): (a) 10,000 versus 1,000 and (b) 10,000 versus 100,000. (c,d) Comparison of estimated parameters of distributions of of microbial assemblages over genera (indicated as $\phi_{k,j}$ in the main text): (c) 10,000 versus 1,000 and (d) 10,000 versus 100,000. The $x$- and $y$-axes represent the estimated values of parameters in the cases of 1,000/10,0000 and 10,000, respectively.

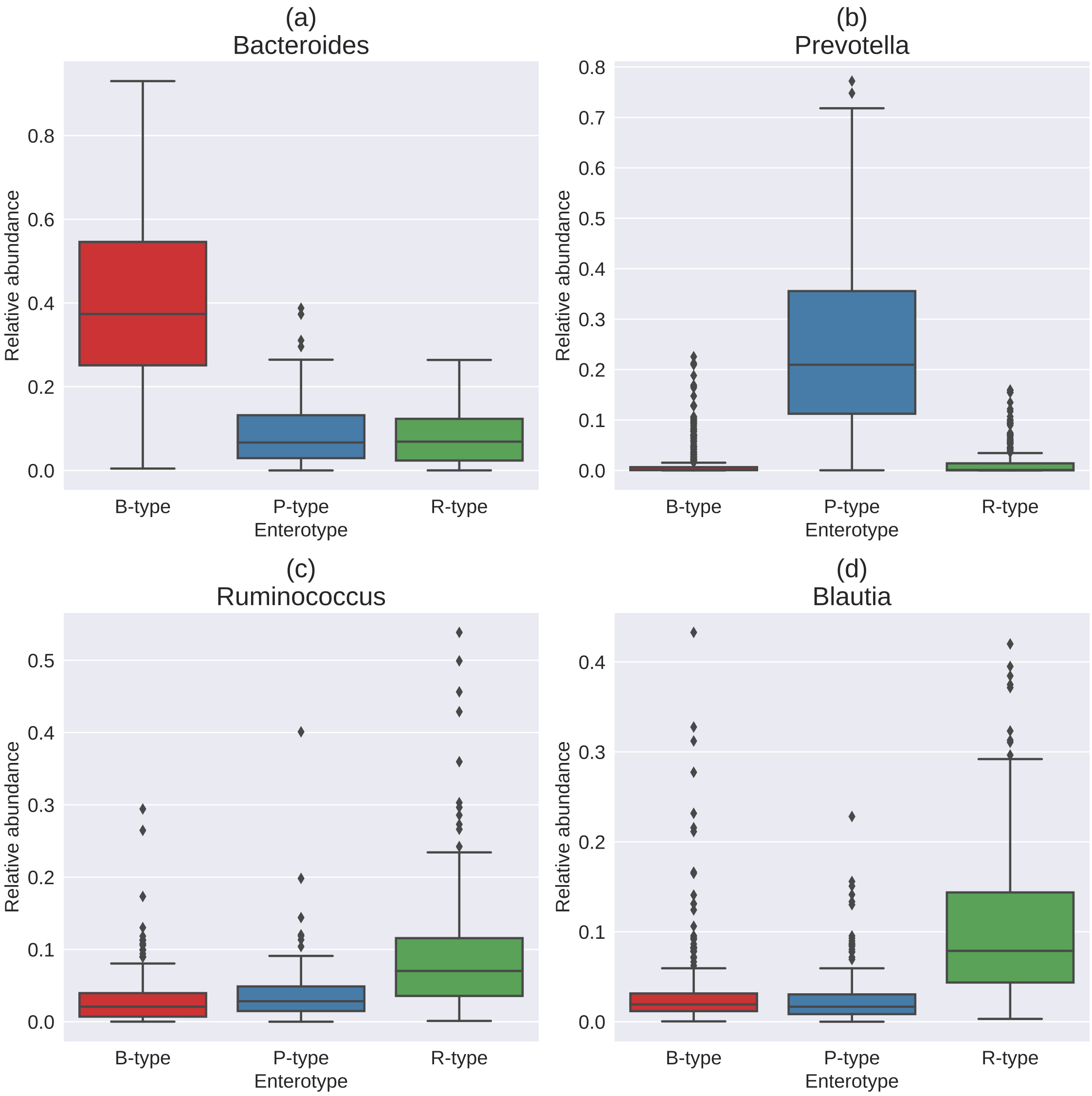


Figure S2: The relative abundance of four major genera ((a)*Bacteroides*, (b)*Prevotella*, (c)*Ruminococcus*, (d)*Blautia*) for each enterotype. The $x$- and $y$-axes represent the enterotype and relative abundance of the genus, respectively.

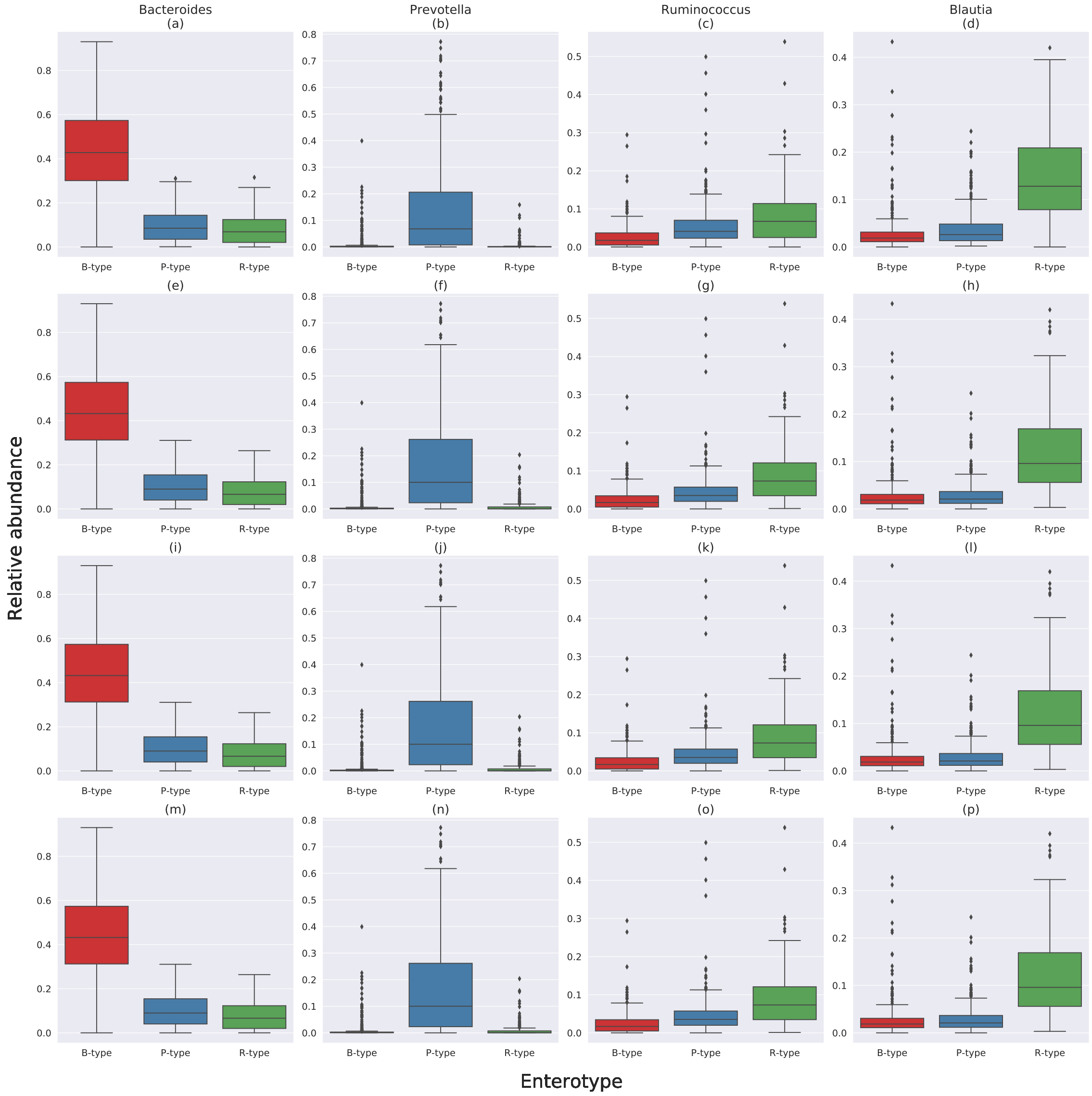


Figure S3: Relative abundance of four major genera ( (a, e, i, m) *Bacteroides*, (b, f, j, n) *Prevotella*, (c, g, k, o) *Ruminococcus*, (d, h, l, p) *Blautia*) for each enterotype obtained by four trials with the (a-d) second, (e-h) third, (i-l) fourth, and (m-p) fifth highest silhouette coefficients. The $x$- and $y$-axes represent enterotype and relative abundance of the genus, respectively.

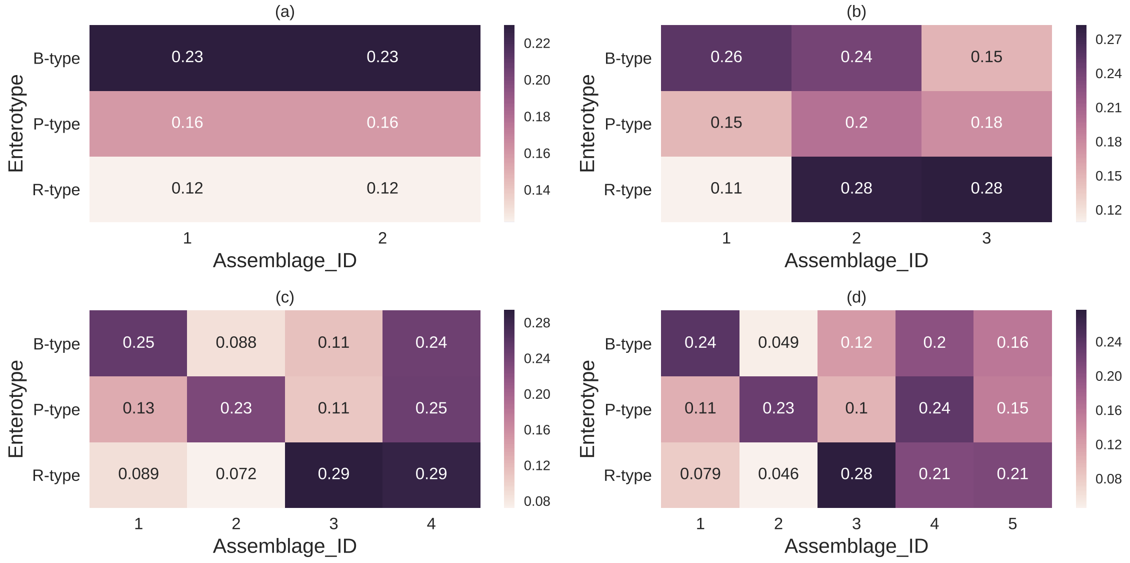


Figure S4: Standard deviations of the assemblage distributions for each enterotype. The $x$- and $y$-axes represent the microbial assemblages and enterotypes, respectively. Darker colors indicate higher standard deviations, and each number inside the partition indicates a different standard deviation. (a), (b), (c), and (d) indicate $K$-assemblage LDA models with $K = 2, 3, 4,$ and 5, respectively.

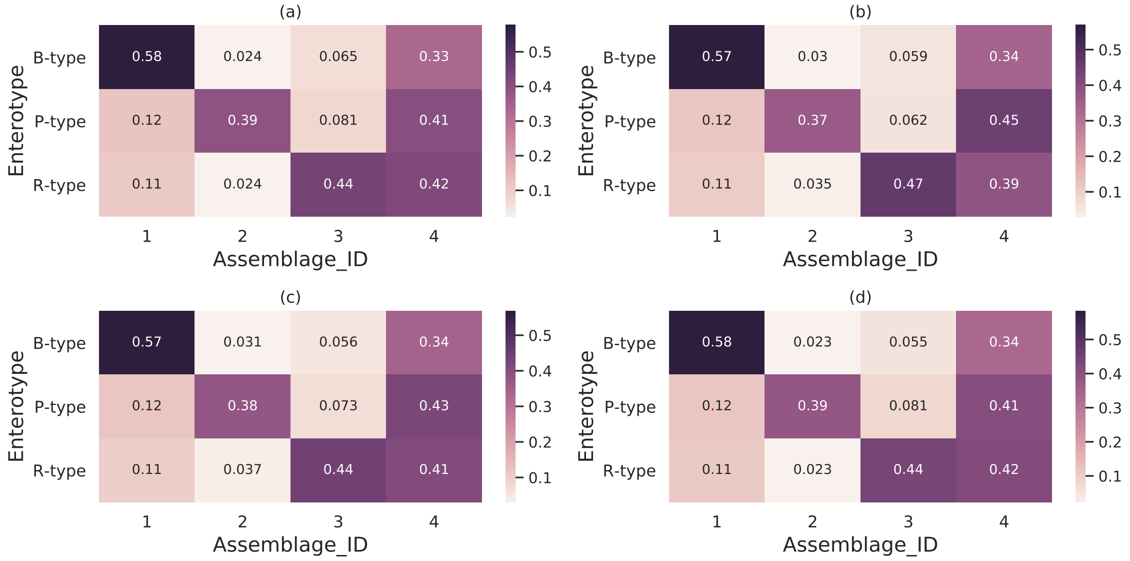


Figure S5: Assemblage distributions of the other four trials with the second, third, fourth, and fifth highest VLB scores for each enterotype. Each row shows a distribution obtained by averaging the estimated assemblage distributions across individuals corresponding to each enterotype. The $x$- and $y$-axes represent the microbial assemblages and enterotypes, respectively. Darker colors indicate higher probabilities, and each number inside the partition indicates a different probability, where the sum of the values over each row is 1. (a), (b), (c), and (d) indicate the trials with the second, third, fourth, and fifth highest VLB scores, respectively.

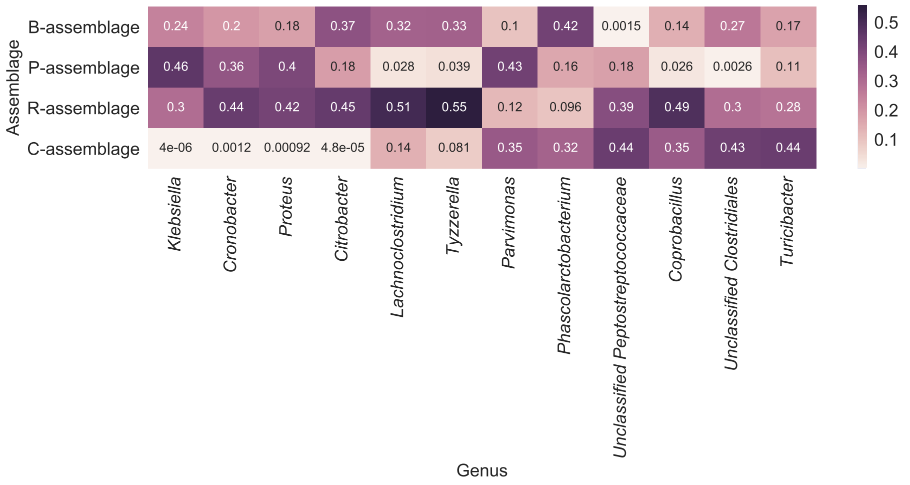


Figure S6: The $P(k|j)$ (Eq. 3 in the main text) of the genera with high entropy scores ($> 1.0$). The $x$- and $y$-axes represent the genus and microbial assemblage, respectively.

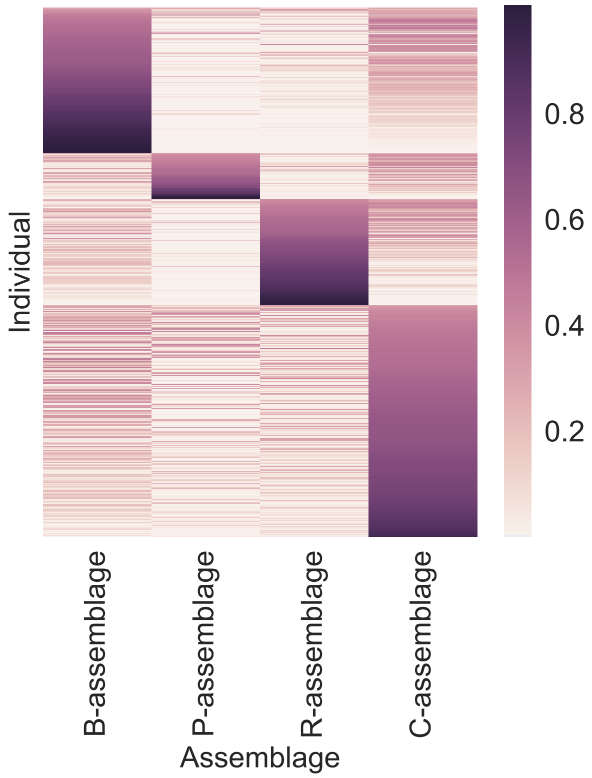


Figure S7: Estimated microbial assemblage distribution for each individual. The $x$- and $y$-axes represent microbial assemblages and individual samples, respectively. Individuals are ordered by the probability of the dominant assemblage.

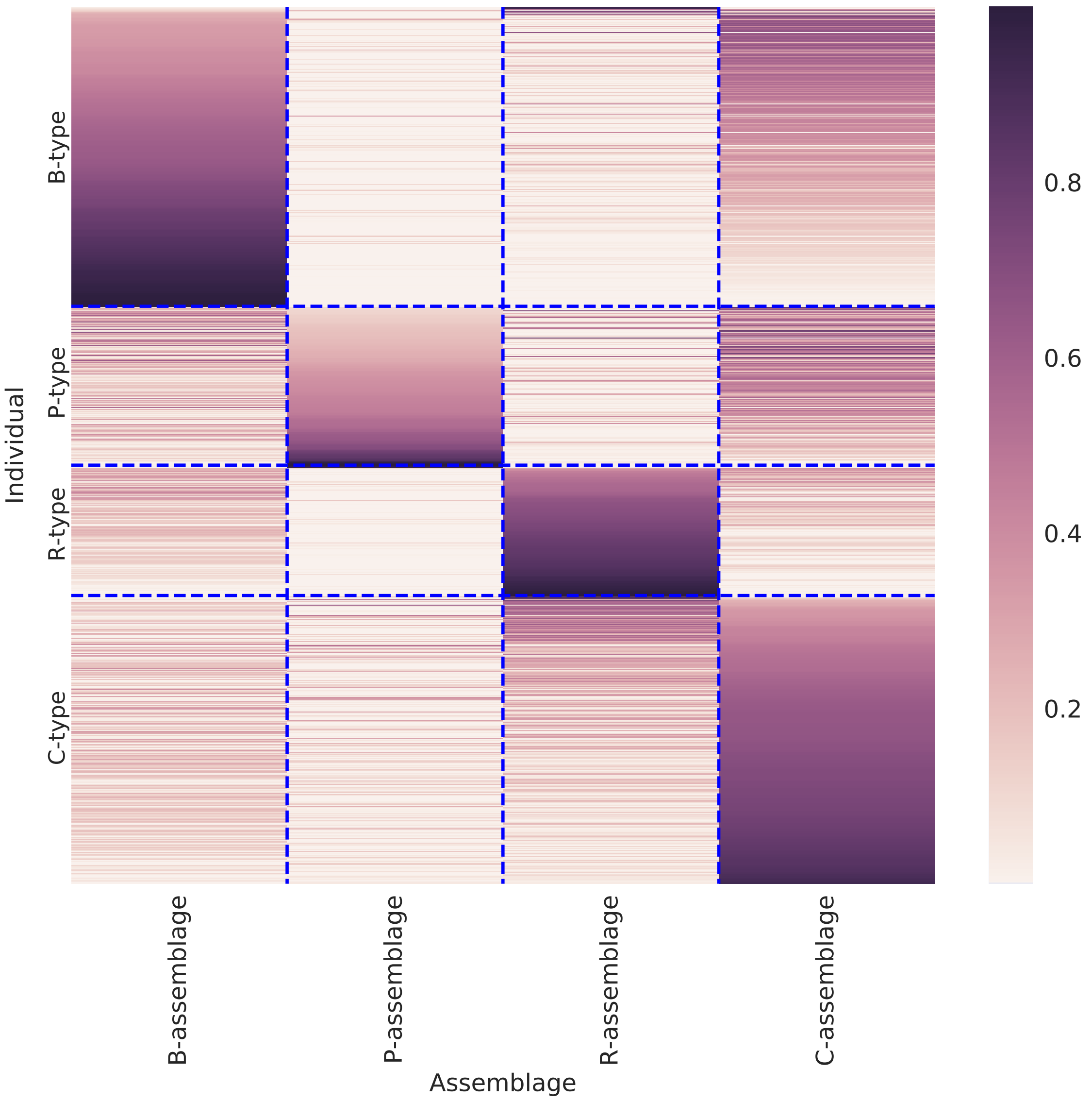


Figure S8: Estimated microbial assemblage distribution for each individual. The $x$- and $y$-axes represent microbial assemblages and individual samples, respectively. Individuals are segregated by the types obtained by the 4-type PAM method and sorted by the B-assemblage, P-assemblage, R-assemblage, and C-assemblage, respectively. The fourth type is referred to as the “C-type.”

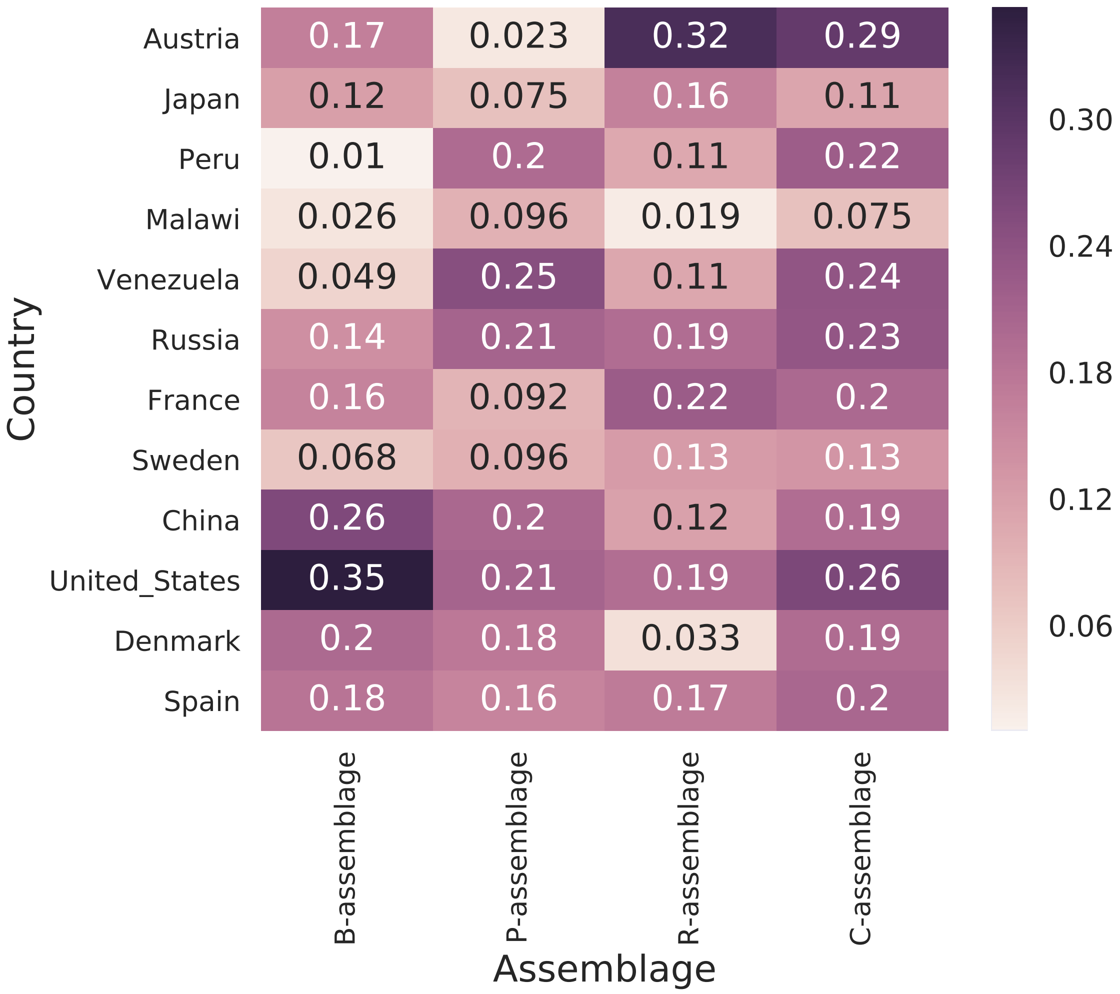


Figure S9: Standard deviations of the assemblage distributions for each country. The $x$- and $y$-axes represent the microbial assemblages and countries, respectively. Darker colors indicate higher standard deviations, and each number inside the partition indicates a different standard deviation. (a), (b), (c), and (d) indicate $K$-assemblage LDA models with $K = 2, 3, 4,$ and 5, respectively.

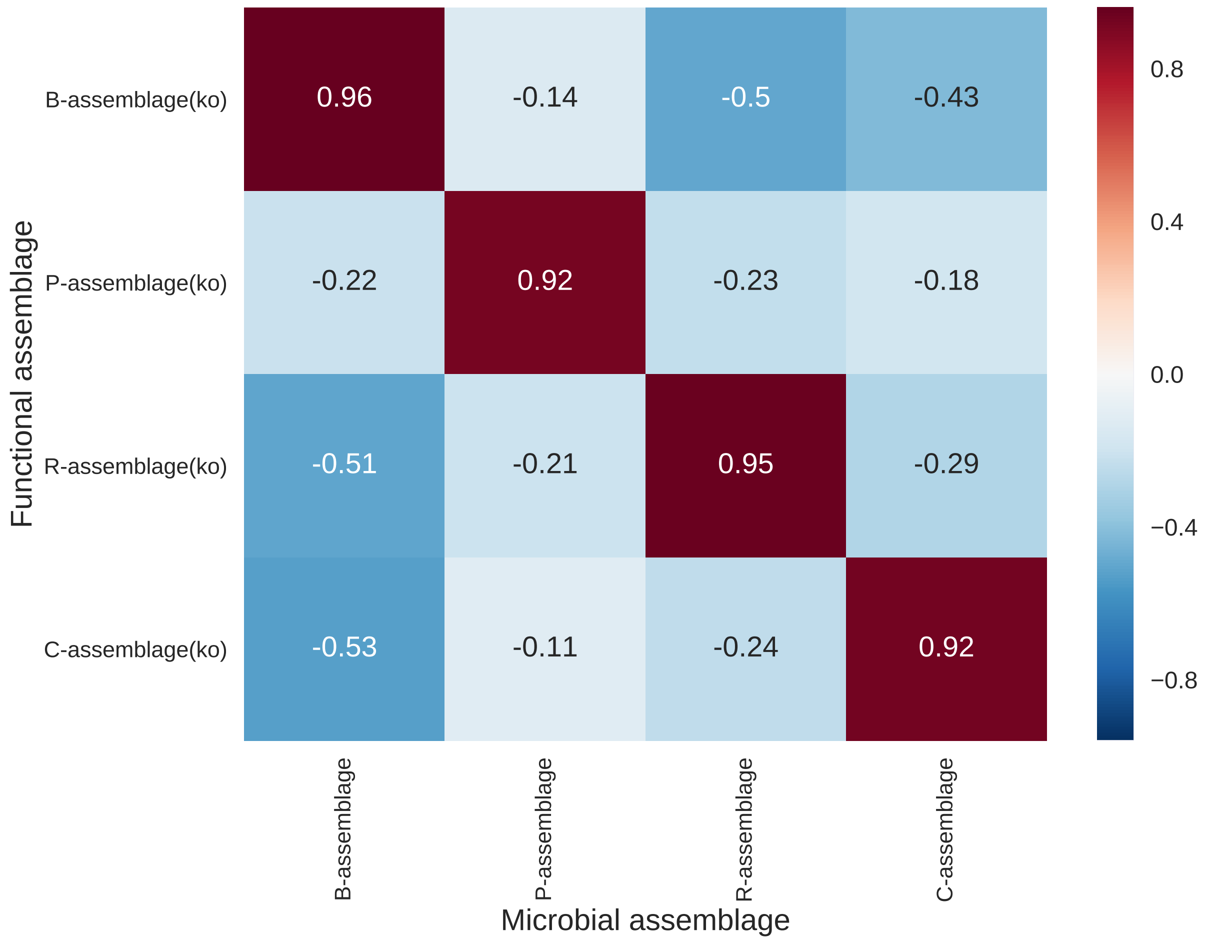


Figure S10: Pearson's correlation coefficients among microbial and functional assemblages (the same figure as Fig. A2(3)). Both the $x$- and $y$-axes represent assemblages.

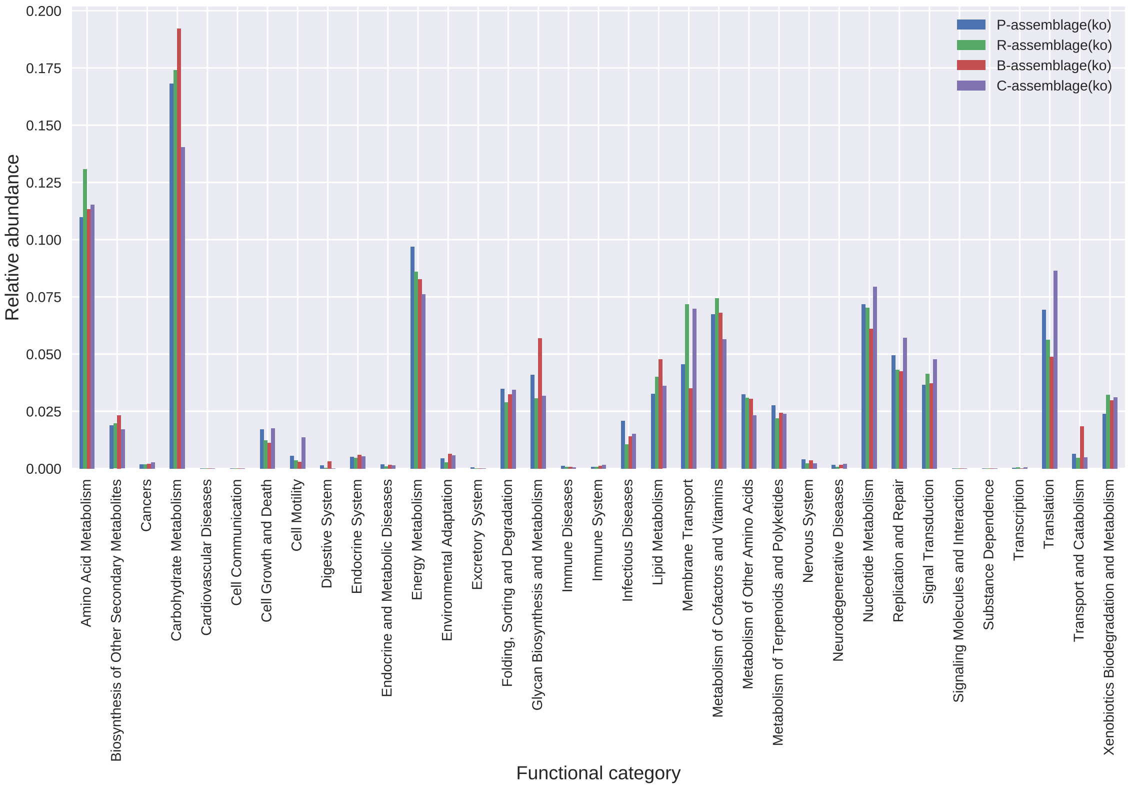


Figure S11: Relative abundance of functional categories for each assemblage. The $x$- and $y$-axes represent the function category and relative abundance, respectively.

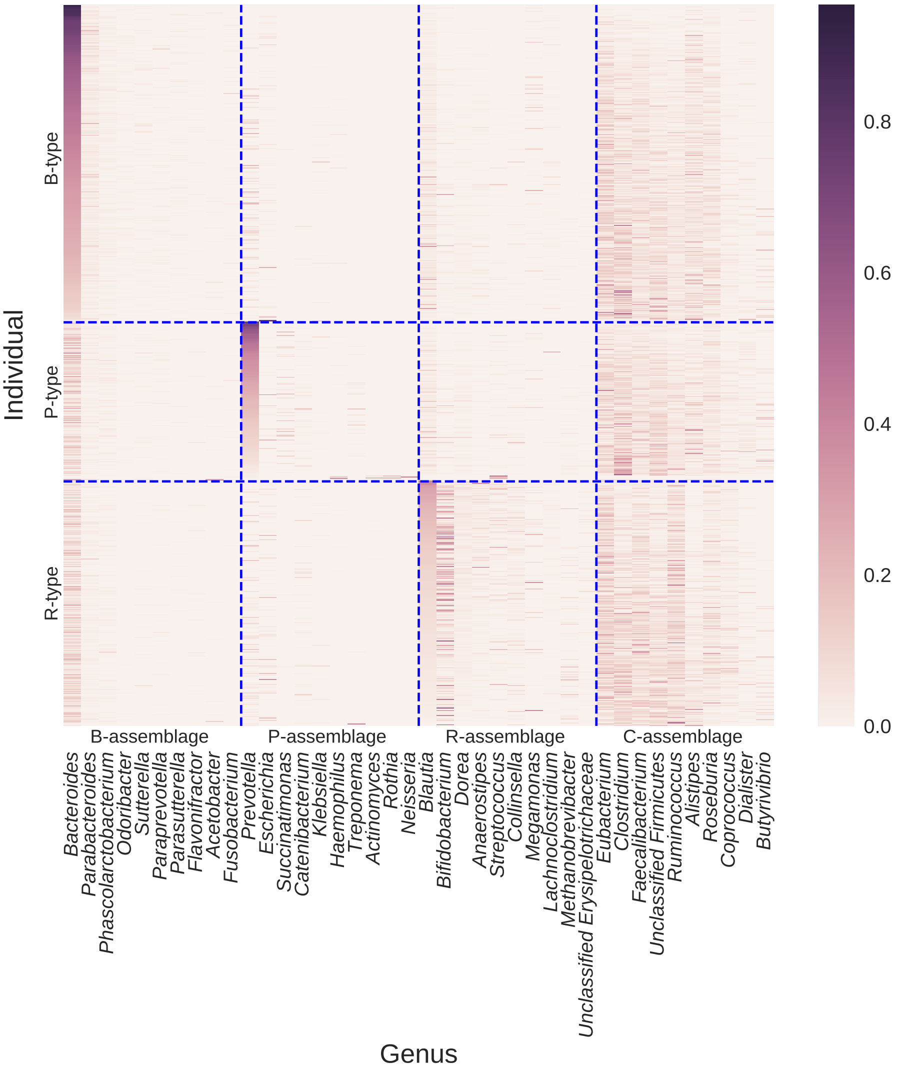


Figure S12: Relative abundance of genera in each individual. The $x$- and $y$-axes represent the genus and individuals, respectively. Individuals are divided by the enterotype and sorted by *Bacteroides*, *Prevotella*, and *Blautia*. Genera are divided by the assemblage in which they mainly appear and are sorted by the abundance of each genus. Each genus was regarded as mainly appearing in the assemblage of the highest $P(k|j)$, which is a probability of the $k$-th assemblage given the $j$-th genus (Eq. 3 in the main text).

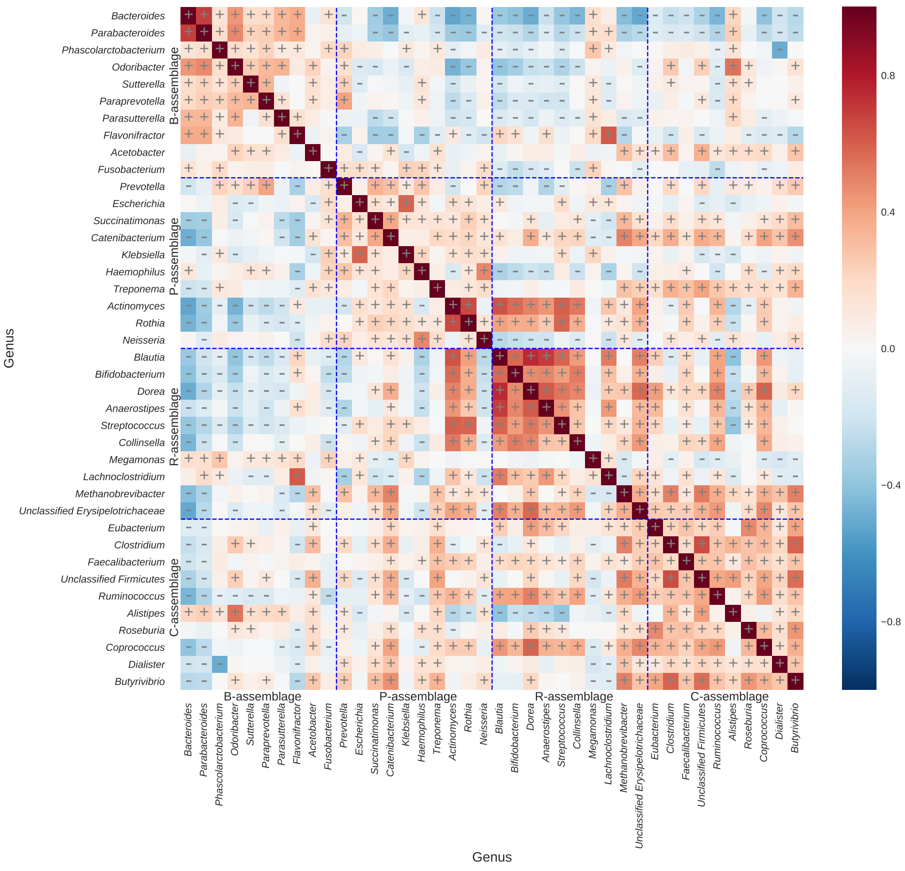


Figure S13: The Spearman's correlation coefficients among the 20 genera that are predominant in each enterotype. Both the $x$- and $y$-axes represent genera, which are divided and sorted similarly as in Fig. S12. Plus and minus signs indicate significant positive and negative correlations, respectively. Significance was determined at $p < 0.01$ (two-sided test, after Benjamini–Hochberg correction).

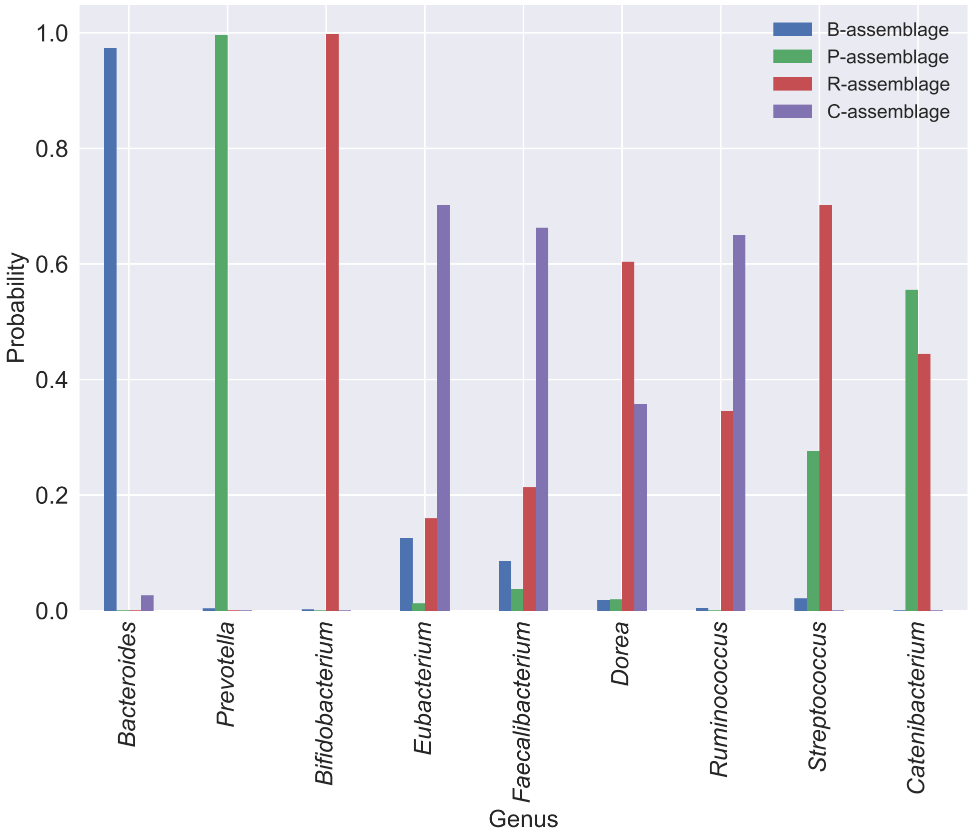


Figure S14: $P(k|j)$ of *Bacteroides*, *Prevotella*, *Bifidobacterium*, and the genera that have high Spearman's correlation coefficients with the other assemblage genera in Fig. S13. The $x$- and $y$-axes represent the genus and $P(k|j)$, respectively.

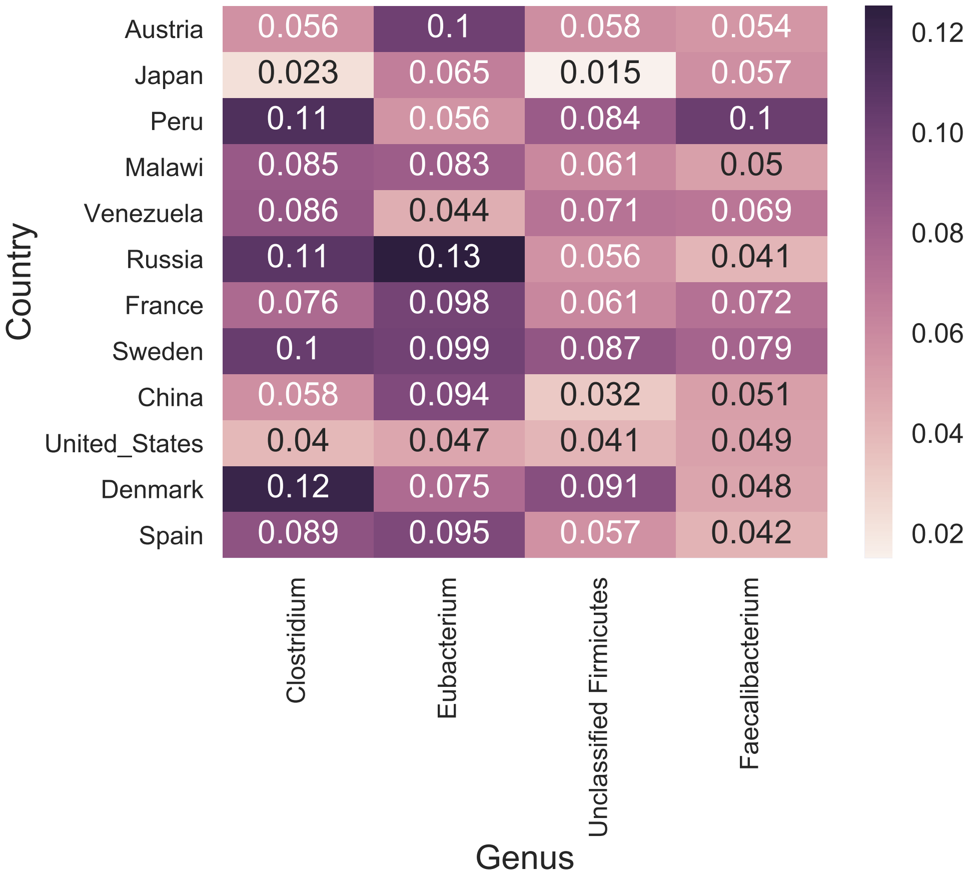


Figure S15: Average relative abundance of *Clostridium*, *Eubacterium*, unclassified *Firmicutes*, and *Faecalibacterium* for each country. The $x$- and $y$-axes represent the genus and country of the individual, respectively.

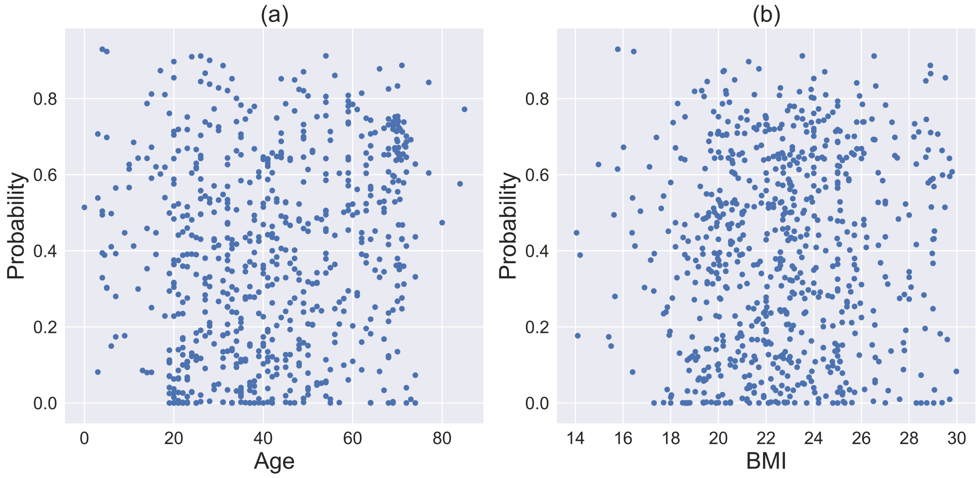


Figure S16: Relationship between C-assemblage probability and (a) age and (b) BMI metadata. The $x$- and $y$-axes represent the metadata and C-assemblage probability, respectively.

Table S1: The entropy score of $P(k|j)$ for all genera used.

| Genus | entropy |
|---|---|
| *Turicibacter* | 1.260681 |
| *Phascolarctobacterium* | 1.245466 |
| *Parvimonas* | 1.220488 |
| *Unclassified Clostridiales* | 1.092140 |
| *Coprobacillus* | 1.088414 |
| *Lachnoclostridium* | 1.082888 |
| *Klebsiella* | 1.061892 |
| *Cronobacter* | 1.056325 |
| *Unclassified Peptostreptococcaceae* | 1.044608 |
| *Proteus* | 1.042833 |
| *Citrobacter* | 1.037434 |
| *Tyzzerella* | 1.021686 |
| *Veillonella* | 0.999699 |
| *Holdemania* | 0.983926 |
| *Yersinia* | 0.980842 |
| *Bilophila* | 0.951916 |
| *Faecalibacterium* | 0.937553 |
| *Unclassified Lachnospiraceae* | 0.933686 |
| *Paraprevotella* | 0.926816 |
| *Enterobacter* | 0.924527 |
| *Unclassified Bacteria* | 0.906336 |
| *Subdoligranulum* | 0.893402 |
| *Pseudomonas* | 0.891144 |
| *Peptoclostridium* | 0.880690 |
| *Flavonifractor* | 0.878429 |
| *Eubacterium* | 0.857729 |
| *Lachnospira* | 0.840097 |
| *Dorea* | 0.822750 |
| *Unclassified Erysipelotrichaceae* | 0.816711 |
| *Escherichia* | 0.814104 |
| *Megasphaera* | 0.812753 |
| *Roseburia* | 0.788690 |
| *Erysipelatoclostridium* | 0.784843 |
| *Acidaminococcus* | 0.773040 |
| *Dielma* | 0.756564 |
| *Intestinibacter* | 0.744818 |
| *Mycobacterium* | 0.736164 |
| *Sutterella* | 0.725791 |
| *Scardovia* | 0.718660 |
| *Olsenella* | 0.713303 |
| *Providencia* | 0.709675 |
| *Stomatobaculum* | 0.709085 |
| *Odoribacter* | 0.704806 |
| *Unclassified Clostridiales Family XIII. Incerta...* | 0.703168 |
| *Peptostreptococcus* | 0.697437 |
| *Kandleria* | 0.696656 |
| *Marvinbryantia* | 0.695373 |
| *Cloacibacillus* | 0.695198 |
| *Sharpea* | 0.695112 |
| *Atopobium* | 0.694374 |
| *Barnesiella* | 0.693038 |
| *Catenibacterium* | 0.687024 |
| *Tannerella* | 0.686389 |
| *Streptococcus* | 0.685115 |
| *Oenococcus* | 0.684101 |
| *Unclassified Bacteroidales* | 0.683936 |

Table S1: The entropy score of $P(k|j)$ for all genera used. – *Continued from previous page*

| Genus | entropy |
|---|---|
| *Dialister* | 0.683460 |
| *Azospirillum* | 0.680177 |
| *Weissella* | 0.677471 |
| *Ruminococcus* | 0.673173 |
| *Megamonas* | 0.669763 |
| *Leuconostoc* | 0.668794 |
| *Unclassified Burkholderiales* | 0.665163 |
| *Methanosphaera* | 0.654763 |
| *Blautia* | 0.646975 |
| *Solobacterium* | 0.644240 |
| *Enterococcus* | 0.644030 |
| *Akkermansia* | 0.631433 |
| *Alistipes* | 0.624308 |
| *Actinomyces* | 0.622316 |
| *Unclassified Ruminococcaceae* | 0.620387 |
| *Peptoniphilus* | 0.616613 |
| *Senegalimassilia* | 0.595117 |
| *Pseudoflavonifractor* | 0.594075 |
| *Unclassified Proteobacteria* | 0.591897 |
| *Haemophilus* | 0.588196 |
| *Desulfovibrio* | 0.585395 |
| *Alloprevotella* | 0.582315 |
| *Actinobaculum* | 0.579283 |
| *Propionibacterium* | 0.578085 |
| *Oxalobacter* | 0.576018 |
| *Mitsuokella* | 0.571258 |
| *Coprococcus* | 0.562809 |
| *Fusobacterium* | 0.561115 |
| *Adlercreutzia* | 0.546762 |
| *Butyricicoccus* | 0.543292 |
| *Anaerostipes* | 0.539088 |
| *Thauera* | 0.535747 |
| *Pedobacter* | 0.535627 |
| *Luteimonas* | 0.535625 |
| *Allobaculum* | 0.535624 |
| *Sphaerochaeta* | 0.535624 |
| *Kallipyga* | 0.535624 |
| *Lamprocystis* | 0.535565 |
| *Sphingobacterium* | 0.535565 |
| *Sphingopyxis* | 0.535565 |
| *Unclassified Aminicenantes* | 0.535499 |
| *Brevibacillus* | 0.535499 |
| *Campylobacter* | 0.521678 |
| *Methanobrevibacter* | 0.520127 |
| *Cryptobacterium* | 0.513993 |
| *Ruminiclostridium* | 0.510680 |
| *Eggerthella* | 0.509646 |
| *Lactococcus* | 0.504261 |
| *Butyricimonas* | 0.491595 |
| *Unclassified Bacteroidetes* | 0.474846 |
| *Shewanella* | 0.459807 |
| *Porphyromonas* | 0.434124 |
| *Parabacteroides* | 0.427271 |
| *Johnsonella* | 0.423664 |
| *Corynebacterium* | 0.420475 |

Table S1: The entropy score of $P(k|j)$ for all genera used. – *Continued from previous page*

| Genus | entropy |
|---|---|
| *Brachyspira* | 0.393297 |
| *Gemella* | 0.377228 |
| *Gordonibacter* | 0.369317 |
| *Lactobacillus* | 0.347828 |
| *Unclassified Xanthomonadaceae* | 0.339638 |
| *Dickeya* | 0.339637 |
| *Kosakonia* | 0.339551 |
| *Moraxella* | 0.339550 |
| *Arthrospira* | 0.339549 |
| *Anaeroglobus* | 0.339549 |
| *Carnobacterium* | 0.339549 |
| *Shinella* | 0.339507 |
| *Stenotrophomonas* | 0.339459 |
| *Allofustis* | 0.339458 |
| *Lelliottia* | 0.339458 |
| *Bacillus ¡bacterium¿* | 0.336655 |
| *Hafnia* | 0.325478 |
| *Granulicatella* | 0.298182 |
| *Collinsella* | 0.294826 |
| *Actinobacillus* | 0.253157 |
| *Mobiluncus* | 0.253157 |
| *Cupriavidus* | 0.253089 |
| *Unclassified Propionibacteriaceae* | 0.253089 |
| *Shuttleworthia* | 0.253055 |
| *Ethanoligenens* | 0.253055 |
| *Finegoldia* | 0.253018 |
| *Shigella* | 0.252294 |
| *Coprobacter* | 0.251584 |
| *Rikenella* | 0.240003 |
| *Parasutterella* | 0.239172 |
| *Lachnoanaerobaculum* | 0.235800 |
| *Clostridium* | 0.216568 |
| *Sinorhizobium* | 0.203630 |
| *Aeromicrobium* | 0.203630 |
| *Tetrasphaera* | 0.203574 |
| *Janthinobacterium* | 0.203572 |
| *Dysgonomonas* | 0.203572 |
| *Microlunatus* | 0.203546 |
| *Pantoea* | 0.203546 |
| *Synergistes* | 0.203514 |
| *Unclassified Firmicutes* | 0.174778 |
| *Vibrio* | 0.171243 |
| *Anaerovibrio* | 0.171171 |
| *Bulleidia* | 0.171171 |
| *Unclassified Oxalobacteraceae* | 0.171171 |
| *Lachnobacterium* | 0.171144 |
| *Raoultella* | 0.169913 |
| *Abiotrophia* | 0.169097 |
| *Anaerotruncus* | 0.168921 |
| *Streptomyces* | 0.157833 |
| *Thermus* | 0.148239 |
| *Trueperella* | 0.148238 |
| *Serratia* | 0.148238 |
| *Macrococcus* | 0.148238 |
| *Terrisporobacter* | 0.148238 |

Table S1: The entropy score of $P(k|j)$ for all genera used. – *Continued from previous page*

| Genus | entropy |
|---|---|
| *Parascardovia* | 0.148238 |
| *Paenibacillus* | 0.136984 |
| *Riemerella* | 0.131086 |
| *Sphingomonas* | 0.131048 |
| *Candidatus Arthromitus* | 0.131048 |
| *Negativicoccus* | 0.131030 |
| *Bacteroides* | 0.122743 |
| *Candidatus Stoquefichus* | 0.117656 |
| *Aggregatibacter* | 0.112499 |
| *Pseudoalteromonas* | 0.106937 |
| *Unclassified Betaproteobacteria* | 0.106937 |
| *Robinsoniella* | 0.106905 |
| *Filifactor* | 0.106891 |
| *Brevibacterium* | 0.106891 |
| *Kocuria* | 0.098099 |
| *Unclassified Alphaproteobacteria* | 0.098099 |
| *Georgenia* | 0.098099 |
| *Mogibacterium* | 0.098071 |
| *Paracoccus* | 0.098056 |
| *Morganella* | 0.090671 |
| *Rothia* | 0.089105 |
| *Nocardioides* | 0.084365 |
| *Staphylococcus* | 0.082676 |
| *Unclassified Coriobacteriaceae* | 0.078953 |
| *Anaerofustis* | 0.078953 |
| *Slackia* | 0.078174 |
| *Pasteurella* | 0.070096 |
| *Aerococcus* | 0.070047 |
| *Anaerococcus* | 0.066380 |
| *Anaerosalibacter* | 0.057431 |
| *Unclassified Candidatus Saccharibacteria* | 0.057431 |
| *Gardnerella* | 0.052776 |
| *Plesiomonas* | 0.050727 |
| *Xanthomonas* | 0.048869 |
| *Elizabethkingia* | 0.048838 |
| *Succinimonas* | 0.047110 |
| *Selenomonas* | 0.046316 |
| *Oribacterium* | 0.044003 |
| *Beggiatoa* | 0.041308 |
| *Unclassified Bacteroidaceae* | 0.040074 |
| *Acidovorax* | 0.035891 |
| *Bifidobacterium* | 0.035527 |
| *Micrococcus* | 0.029757 |
| *Catonella* | 0.029757 |
| *Aeromonas* | 0.028547 |
| *Leclercia* | 0.026936 |
| *Kluyvera* | 0.026434 |
| *Burkholderia* | 0.025968 |
| *Geobacillus* | 0.025960 |
| *Alloscardovia* | 0.025499 |
| *Rhodococcus* | 0.025052 |
| *Prevotella* | 0.024904 |
| *Microbacterium* | 0.024628 |
| *Acinetobacter* | 0.023983 |
| *Varibaculum* | 0.023434 |

Table S1: The entropy score of $P(k|j)$ for all genera used. – *Continued from previous page*

| Genus | entropy |
|---|---|
| *Listeria* | 0.022350 |
| *Alcanivorax* | 0.022160 |
| *Eikenella* | 0.020773 |
| *Oscillibacter* | 0.020362 |
| *Enterorhabdus* | 0.018686 |
| *Helicobacter* | 0.017187 |
| *Bifidobacterium* | 0.013555 |
| *Methylobacterium* | 0.012762 |
| *Leptotrichia* | 0.012559 |
| *Pyramidobacter* | 0.010146 |
| *Lysinibacillus* | 0.009544 |
| *Kingella* | 0.008453 |
| *Bordetella* | 0.007775 |
| *Cardiobacterium* | 0.007596 |
| *Methanomassiliicoccus* | 0.006476 |
| *Lautropia* | 0.005972 |
| *Succinatimonas* | 0.004608 |
| *Spiroplasma* | 0.004588 |
| *Acidiphilium* | 0.003029 |
| *Corallococcus* | 0.002747 |
| *Achromobacter* | 0.002647 |
| *Capnocytophaga* | 0.002549 |
| *Cetobacterium* | 0.002347 |
| *Butyrivibrio* | 0.002281 |
| *Coraliomargarita* | 0.002105 |
| *Enorma* | 0.001869 |
| *Acholeplasma* | 0.000901 |
| *Pediococcus* | 0.000880 |
| *Neisseria* | 0.000365 |
| *Treponema* | 0.000171 |
| *Acetobacter* | 0.000142 |
